# Estrogen receptor β deficiency increases susceptibility to sepsis through metabolic reprogramming–induced macrophage pyroptosis

**DOI:** 10.1172/JCI196636

**Published:** 2026-03-17

**Authors:** Yanrong Zhu, Gang Li, Yilei Guo, Yue He, Wanyi Zhang, Lei Gao, Jing Zhang, Pengxiang Guo, Haochang Lin, Wenjie Zhang, Zhifeng Wei, Yufeng Xia, Yue Dai

**Affiliations:** 1 Department of Pharmacology of Chinese Materia Medica, School of Traditional Chinese Pharmacy, China Pharmaceutical University, Nanjing, China.; 2 Department of Emergency Medicine, The Third Affiliated Hospital of Zhejiang Chinese Medical University, Hangzhou, Zhejiang, China.; 3 Department of Pharmacognosy, School of Traditional Chinese Pharmacy, China Pharmaceutical University, Nanjing, China.

**Keywords:** Immunology, Inflammation, Macrophages

## Abstract

Understanding susceptibility factors of sepsis is crucial for early diagnosis and development of personalized treatment strategies. However, the genetic determinants for initiation and progression of sepsis remain unclear. Here, we showed that the expression levels of estrogen receptor β (ERβ) were significantly reduced in the peripheral blood of patients with sepsis and were negatively correlated with disease severity. The results from human samples and experimental animals demonstrated that ERβ deficiency enhanced the body’s susceptibility to sepsis by inducing macrophage pyroptosis, thereby impairing bacterial clearance. Mechanistically, ERβ deficiency enhanced fatty acid oxidation, increased acetyl-CoA levels, and promoted acetylation of stomatin-like protein 2 (Stoml2) at K221, leading to mitochondrial dysfunction and macrophage pyroptosis. Mutating the Stoml2 K221 site mitigated these effects and improved survival of septic mice. These findings suggest ERβ deficiency as a potential genetic factor in sepsis susceptibility.

## Abstract 2

ERβ is a susceptibility gene for sepsis, and its deficiency worsens sepsis outcomes through metabolic reprogramming induced macrophage pyroptosis.

## Graphical abstract

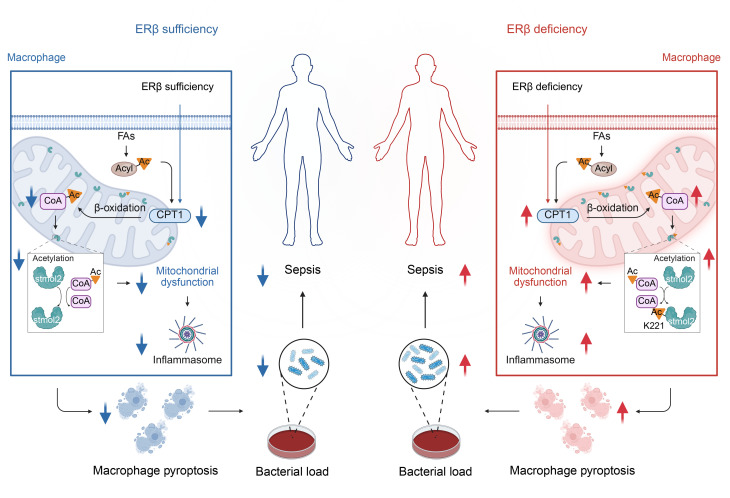

## Introduction

Sepsis, a life-threatening syndrome characterized by acute organ dysfunction resulting from a dysregulated response to infection, commonly originates from the lungs, urinary tract, and abdomen (1–3). Worldwide, an estimated 48.9 million cases of sepsis and 11 million related deaths occur annually (4). In the United States, more than one-third of in-hospital deaths are attributed to sepsis, with costs exceeding $38 billion in 2017, making it both the most common cause of in-hospital death and the most expensive cause of hospitalization (5). Although the pathogenesis of sepsis is still not fully understood, genetic susceptibility is recognized to be closely related to its occurrence and development (5). The differential expression of genes in sepsis has long confounded clinicians, presenting substantial obstacles to timely diagnosis and effective treatment (6). Carriers of *TNF2*, who exhibit excessive production of TNF-α, experience marked higher mortality even when receiving standard treatment (7). Individuals carrying IL1RN*2 are more susceptible to sepsis, with a substantially increased risk of sepsis-related mortality (8). However, the IL-1 receptor antagonist anakinra, which targets IL-1β, has shown survival benefits in cases of septic shock and COVID-19–associated sepsis and has been FDA approved for treating patients with COVID-19 (9, 10). Therefore, the discovery of genetic susceptibility factors in sepsis constitutes an important area of research that should provide effective treatment strategies for sepsis (11).

The incidence and mortality rates of sepsis exhibit a sex bias (12). Epidemiological studies reveal that, compared with women, men are more prone to sepsis, and men with sepsis experience higher rates of in-hospital mortality and recurrence (12). Estrogen receptor β (ERβ), the primary estrogen receptor subtype in site-of-origin of sepsis, such as lung and urinary tract, also exhibits sex-biased expression differences in both organs (13–17). Clinical studies have demonstrated that the expression of ERβ is higher in lungs and urinary tracts of women compared with men (16, 17). Furthermore, patients with reduced ERβ expression at the lesion site, such as those with depression, have an increased risk of bloodstream infections and are more susceptible to sepsis-related organ damage (18, 19). In addition, in vivo studies show a marked reduction in ERβ expression in the kidneys and lungs of mice with sepsis-associated multiorgan damage induced by lipopolysaccharide (LPS) (20, 21). Activation of ERβ has been shown to alleviate disease symptoms in septic mice and rats (22, 23). Intravenous administration of ERβ-specific agonists ERB-041 or WAY-202196 or oral gavage of WAY-202196 in mice subjected to cecal ligation and puncture–induced (CLP-induced) sepsis or pneumococcal pneumonia markedly improved disease outcomes, as evidenced by increased survival rates, reduced pathological damage in the small intestine and ileum of CLP mice, and suppressed transcription of multiple pro-inflammatory mediators in pulmonary and ileal tissues (22). Moreover, in the neutropenic rat model of *Pseudomonas aeruginosa* infection, oral administration of WAY-202196 could enhance survival and substantially decrease histopathological scores of the intestinal mucosal surface (23). These findings suggest that ERβ may serve as a susceptibility gene for sepsis. In this study, we investigated the relationship between ERβ expression and susceptibility to sepsis, exploring its potential pathological mechanisms.

## Results

### ERβ expression level is negatively correlated with sepsis severity.

**Figure 1:**
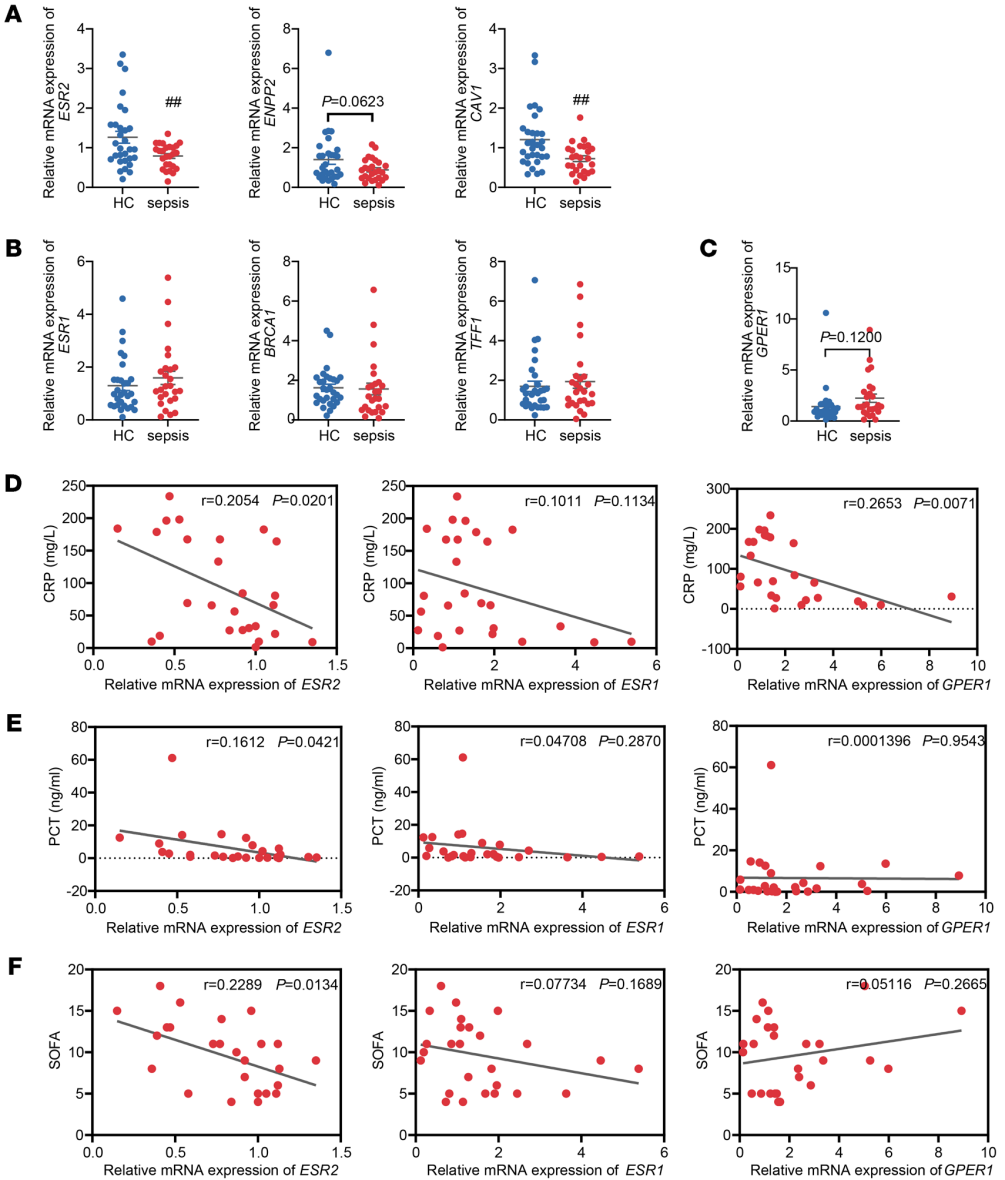
The correlation between ER expression and disease severity in sepsis. Whole blood samples from healthy controls (*n* = 30) and patients with sepsis (*n* = 26) were collected; patients with sepsis were divided into 28-day survivors (*n* = 17) and nonsurvivors (*n* = 9). (**A**) The mRNA expression of *ESR2*, *ENPP2*, and *CAV1* in whole blood samples of healthy controls and patients with sepsis was analyzed using qPCR. (**B**) The mRNA expression of *ESR1*, *BRCA1*, and *TFF1* in whole blood samples of healthy controls and patients with sepsis was analyzed using qPCR. (**C**) The mRNA expression of *GPER1* in whole blood samples of healthy controls and patients with sepsis was analyzed using qPCR. (**D**) Correlation between the mRNA expression of *ESR2*, *ESR1*, and *GPER1* and CRP levels in the blood samples of patients with sepsis was analyzed by Pearson correlation analysis (*n* = 26). (**E**) Correlation between the mRNA expression of *ESR2*, *ESR1*, and *GPER1* and PCT levels in the blood samples of patients with sepsis was analyzed by Pearson’s correlation analysis (*n* = 26). (**F**) Correlation between the mRNA expression of *ESR2*, *ESR1*, and *GPER1* and SOFA of patients with sepsis was analyzed by Pearson’s correlation analysis (*n* = 26). Unpaired Student’s *t* test was performed in **A**–**C**. Data are expressed as mean ± SEM. ^##^*P* < 0.01 versus healthy controls. *r*, Pearson’s correlation coefficient.

To investigate the participation and role of ERβ in sepsis, we first analyzed the expression of *ESR2* and its target genes (*ENPP2* and *CAV1*) in whole blood samples from patients with sepsis and healthy controls. The qPCR results revealed a substantial decrease in the expression of *ESR2* and its downstream target genes in the whole blood of patients with sepsis, compared with healthy controls (Figure 1A). We also examined the expression of 2 other estrogen receptors, ERα and G protein–coupled estrogen receptor (GPER). qPCR results showed no significant differences in the expression of *ESR1* (gene encoding ERα) and its target genes (*BRCA1* and *TFF1*), and *GPER1* (gene encoding GPER) in whole blood samples of patients with sepsis compared with healthy controls (Figure 1, B and C). Subsequently, we performed correlation analysis of *ESR2*, *ENPP2*, and *CAV1* gene expression in whole blood samples from patients with sepsis of varying severity. The results showed that the expression levels of *ESR2* and its targetsʼ genes were negatively correlated with the levels of C-reactive protein (CRP) and procalcitonin (PCT) in the blood samples of patients as well as the sequential organ failure assessment (SOFA) score (Figure 1, D–F, and Supplemental Figure 1; supplemental material available online with this article; https://doi.org/10.1172/JCI196636DS1), but there was no significant correlation between the expression of ERα or its target genes and the severity of the disease (Figure 1, D–F, and Supplemental Figure 1). *GPER1* expression was significantly negatively correlated with the levels of CRP in blood but not with PCT levels or SOFA (Figure 1, D–F). We also divided patients with sepsis into survivors and nonsurvivors according to whether they died at 28 days after admission and also detected *ESR2* and its targetsʼ gene expression in their whole blood samples. Compared with the survivors, the mean expression levels of *ESR2* and its target genes in the whole blood samples of the nonsurvivors were decreased, but there was no significant difference (Supplemental Figure 2). These results indicate that the expression levels of ERβ in the whole blood of sepsis patients are significantly reduced, which are negatively correlated with the severity of the disease. The decrease in ERβ expression may be closely associated with the onset and progression of sepsis, and ERβ deficiency may be a susceptibility factor for sepsis.

### ERβ deficiency worsens sepsis outcomes and impairs bacterial clearance.

**Figure 2:**
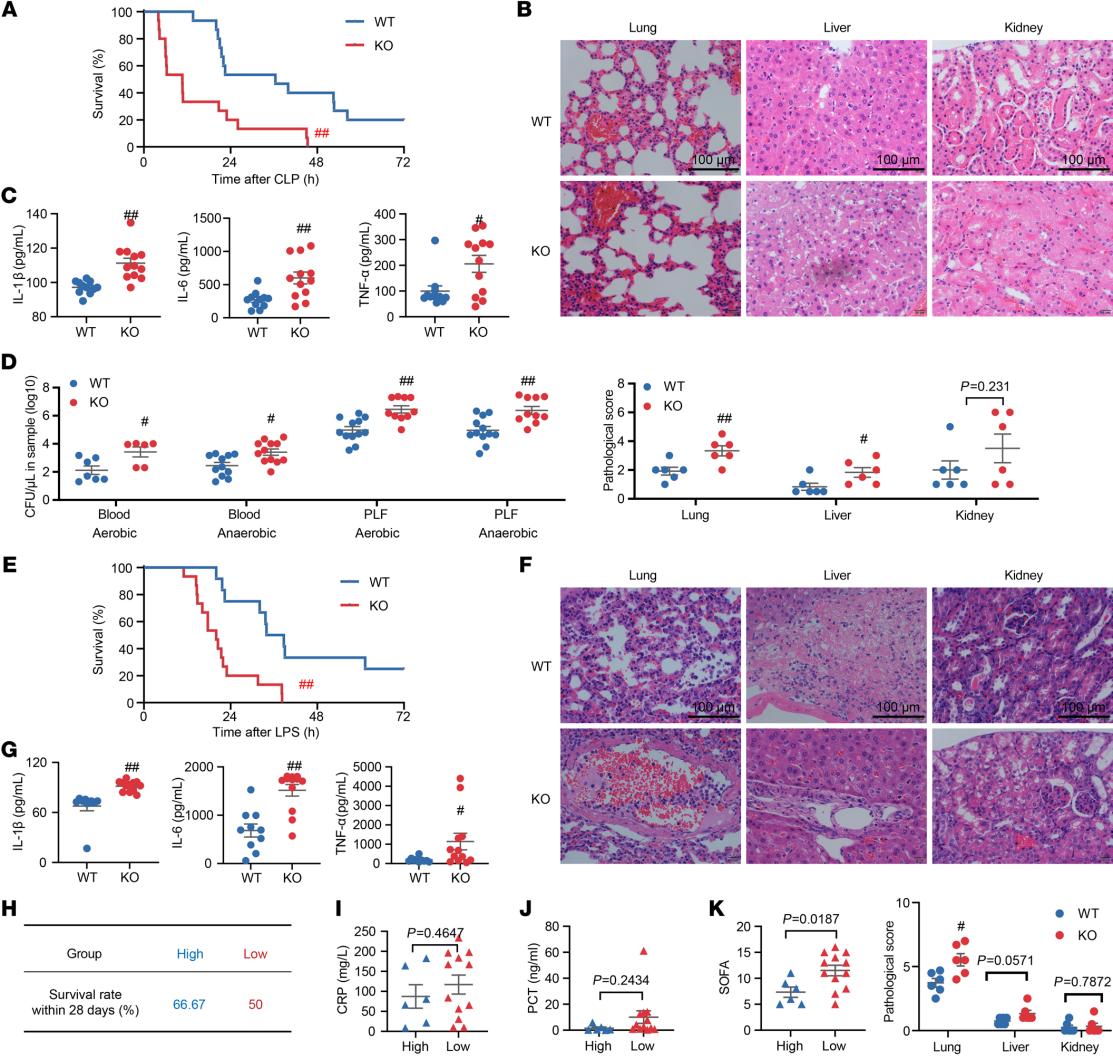
Effect of ERβ deficiency on disease severity in septic mice and patients. (**A**–**D**) The WT and KO mice were subjected to CLP. (**A**) The survival curve of septic mice. *n* = 15. (**B**) H&E staining and histopathological scores of the lung, liver, and kidney from septic mice. *n* = 6. (**C**) Quantification of cytokines in the sera from septic mice. *n* = 11–12. (**D**) Quantification of bacterial colonies in the blood and PLF from septic mice. *n* = 6–12. PLF, peritoneal lavage fluids. (**E**–**G**) The WT and KO mice were subjected to intraperitoneal injection of LPS. (**E**) The survival curve of septic mice. *n* = 12–15. (**F**) H&E staining and histopathological scores of the lung, liver, and kidney from septic mice. *n* = 6. (**G**) Quantification of cytokines in the sera from septic mice. *n* = 10–12. (**H**–**K**) Male patients with sepsis with high *ESR2* expression (high, *n* = 6) and low *ESR2* expression (low, *n* = 12). (**H**) The survival of patients. (**I** and **J**) The levels of CRP (**I**) and PCT (**J**) in blood samples. (**K**) The SOFA of patients. Log-rank (Mantel-Cox) test was adopted to compare the significance in **A** and **E**. Unpaired Student’s *t* test was performed in **B**–**D**, **F**, **G**, and **I**–**K**. Data are expressed as mean ± SEM. ^#^*P* < 0.05 and ^##^*P* < 0.01 versus WT group. Scale bar, 100 μm.

To explore whether ERβ deficiency increases susceptibility to sepsis and aggravates disease symptoms in septic mice, we constructed ERβ-knockout (KO) mice and established the CLP model in both male KO mice and their male littermate wild-type (WT) controls (all subsequent experiments utilized male mice unless otherwise specified). Compared with the WT group, the KO group exhibited a substantial increase in the mortality rate after CLP (Figure 2A). Histopathological examination displayed that the KO mice subjected to CLP developed more severe multiple-organ injury compared with the WT mice (Figure 2B). To further illustrate the effect of ERβ deficiency on the inflammatory response, the concentrations of IL-1β, IL-6, and TNF-α in sera were quantified, and the levels of the 3 cytokines were found to be increased in KO mice (Figure 2C). Live bacteria were increased in the blood and PLF from the KO mice (Figure 2C). Similarly, ERβ deficiency significantly exacerbated disease symptoms in LPS-induced sepsis mice, with reduced survival, increased lung and liver lesions, and upregulated serum levels of IL-1β, IL-6, and TNF-α (Figure 2, E–G). We also established CLP-induced sepsis models in female KO and WT mice. Consistently, we found that ERβ knockout increased susceptibility to sepsis, as evidenced by reduced survival rates, exacerbated histopathological damage, increased bacterial loads in both blood and PLF, and elevated levels of serum pro-inflammatory cytokines (Supplemental Figure 3). The above results indicate that ERβ deficiency significantly increases the susceptibility of mice to sepsis and exacerbates the disease symptoms. Similarly, we examined the impact of ERβ deficiency on the mortality and severity of disease in patients with sepsis. We divided male patients with sepsis into high and low *ESR2* expression groups based on the average *ESR2* expression in healthy controls. Subsequently, a comparison was made of their survival rate, CRP and PCT levels in blood samples, and SOFA score. The results showed that, compared with the patients with high *ESR2* expression, the patients with low *ESR2* expression exhibited a reduced survival rate, an elevated blood PCT level, and a significant elevation in the SOFA score (Figure 2, H–K). Taken together, these results indicate that the deficiency of ERβ enhances the body’s susceptibility to sepsis and exacerbates disease symptoms in both septic patients and mice.

### ERβ deficiency drives macrophage pyroptosis to impair bacterial clearance in sepsis.

**Figure 3:**
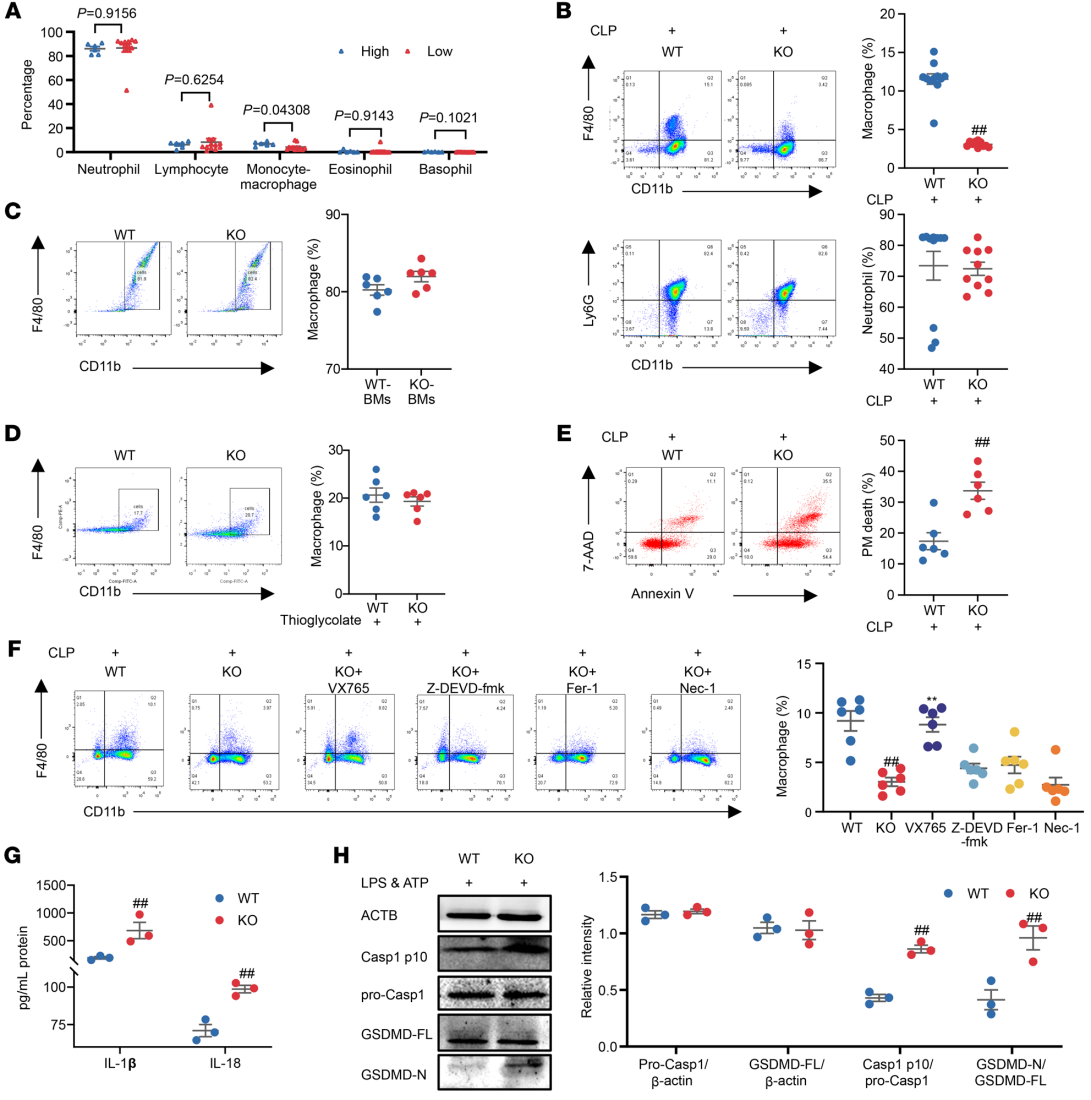
Effect of ERβ deficiency on the proportion of phagocytic cells in the PLF of septic mice induced by CLP and effect of ERβ deficiency on the pyroptosis of primary mouse peritoneal macrophages. (**A**) Male patients with sepsis with high *ESR2* expression (high, *n* = 6) and low *ESR2* expression (low, *n* = 12). The CBC data of male patients with sepsis. (**B**) Flow cytometric analysis of macrophages or neutrophils in PLF from septic mice subjected to CLP. *n* = 10–11. (**C**) Flow cytometric analysis of BM cells from mice. BM cells were treated with GM-CSF (20 ng/mL) for 7 days. *n* = 6. (**D**) Flow cytometric analysis of PMs from mice treated with 3% thioglycolate for 3 days. *n* = 6. (**E**) Cell death analysis of PMs from septic mice at 12 hours after CLP. *n* = 6. (**F**) Flow cytometric analysis of macrophages in PLF from septic mice treated with VX765 (50 mg/kg), ferrostatin-1 (Fer-1, 10 mg/kg), necrostatin-1 (Nec-1, 1.65 mg/kg), or Z-DEVD-FMK (8 mg/kg) (27). *n* = 6. (**G** and **H**) Primary mouse PMs were treated with LPS (2 μg/mL) for 3 hours, followed by ATP (5 mM) treatment for 1 hour. *n* = 3. (**G**) IL-1β and IL-18 secretion was measured in the supernatants of PMs. (**H**) The protein expression of Casp1 p10, pro-Casp1, GSDMD-FL, and GSDMD-N of PMs was analyzed by Western blot. Unpaired Student’s *t* test was performed in **A**–**E**, **G**, and **H**. One-way ANOVA was employed in **F**. Data are expressed as mean ± SEM. ^##^*P* < 0.01 versus WT group; **P < 0.01 versus KO group.

Sepsis-induced mortality is closely associated with the failure to eradicate invading pathogens (24). ERβ deficiency significantly increased bacterial load in the PLF and blood of mice compared with WT controls, prompting us to investigate whether ERβ deficiency influences the body’s bacterial clearance ability (Figure 2D and Supplemental Figure 3, C and D). Phagocytes, including macrophages and neutrophils, play a key role in engulfing and eliminating pathogens (25). To investigate whether ERβ deficiency interferes with pathogen clearance by regulating the proportion of phagocytes, we analyzed the results of complete blood count (CBC) from male septic patients with differential ERβ expression. The results revealed no significant differences in the proportions of neutrophils, lymphocytes, eosinophils, and basophils between sepsis patients with low ERβ expression and those with high ERβ expression (Figure 3A). However, the proportion of monocyte/macrophages was markedly reduced in male sepsis patients with low ERβ expression (Figure 3A). Subsequently, we assessed the proportion of macrophages and neutrophils in the PLF of septic mice, and flow cytometry results showed that ERβ deficiency mice subjected to CLP or LPS treatment exhibited a decrease in the populations of macrophages instead of neutrophils in PLF (Figure 3B and Supplemental Figure 4A). To investigate the mechanism by which ERβ deficiency downregulates macrophage proportions in the context of sepsis, we assessed the impact of ERβ knockout on the ability of bone marrow (BM) cells to differentiate into macrophages, the recruitment of macrophages in the PLF of mice, the proportion of tissue-resident macrophages (TRMs) in the tissue, and the mortality of peritoneal macrophages (PMs) in CLP-induced septic mice (26, 27). We isolated BM cells from WT and KO mice to compare their ability to differentiate into macrophages in response to GM-CSF. Flow cytometry results showed no difference in the macrophage population between these 2 groups (Figure 3C). In response to sterile inflammation induced by thioglycolate, the population of PMs in KO mice showed no difference (Figure 3D), which suggests that ERβ deficiency has no significant effect on macrophage recruitment. Subsequently, we investigated the impact of ERβ on the proportion of TRMs in the PLF of septic mice (26). The results revealed that ERβ deficiency did not exert a significant effect on the proportion of these resident macrophages (Supplemental Figure 4B). We next performed flow cytometry to examine the viability of macrophages and found that ERβ deficiency increased the proportion of cell death of PMs in CLP mice (Figure 3E).

Macrophage death mainly includes apoptosis, pyroptosis, ferroptosis, and necroptosis (27). To identify the primary death mode of macrophages influenced by ERβ, we investigated the effect of 4 death pathway inhibitors on the downregulation of PMsʼ proportions in KO septic mice induced by CLP. Flow cytometry showed that the effect of ERβ deficiency in downregulating the proportion of macrophages in PLF was significantly attenuated by pyroptosis inhibitor VX765 (Figure 3F). In the in vitro experiments, primary mouse PMs were treated with LPS and adenosine triphosphate (ATP) to induce pyroptosis (pyroptosis conditions) (28, 29), and the effect of ERβ deficiency on macrophage pyroptosis was subsequently examined. The results showed that under pyroptosis conditions, ERβ deficiency significantly drove macrophage pyroptosis, as evidenced by the expression upregulation of pyroptosis-related proteins Casp1 p10 and GSDMD-N, increased lactate dehydrogenase (LDH) release, enhanced secretion of IL-1β and IL-18, and facilitated NLRP3 inflammasome assembly, but neither affected the expression of TLR4 or the activation of MyD88-dependent signaling pathway (Figure 3, G and H, and Supplemental Figure 4, D–G). The above results suggest that ERβ deficiency drives macrophage pyroptosis, thereby reducing bacterial clearance ability and ultimately increasing the body’s susceptibility to sepsis.

Given the established involvement of macrophage polarization in the pathogenesis of sepsis, we further quantified the proportions of M1 and M2 macrophage subtypes in the PLF of septic mice (30). The results demonstrated that ERβ deficiency significantly increased the proportion of M1 macrophages but had no significant effect on the proportion of M2 macrophages (Supplemental Figure 4C). These data suggest that the exacerbation of sepsis observed in ERβ-deficient mice may be associated with an altered M1/M2 macrophage balance.

### ERβ deficiency drives macrophage pyroptosis by enhancing fatty acid oxidation.

**Figure 4:**
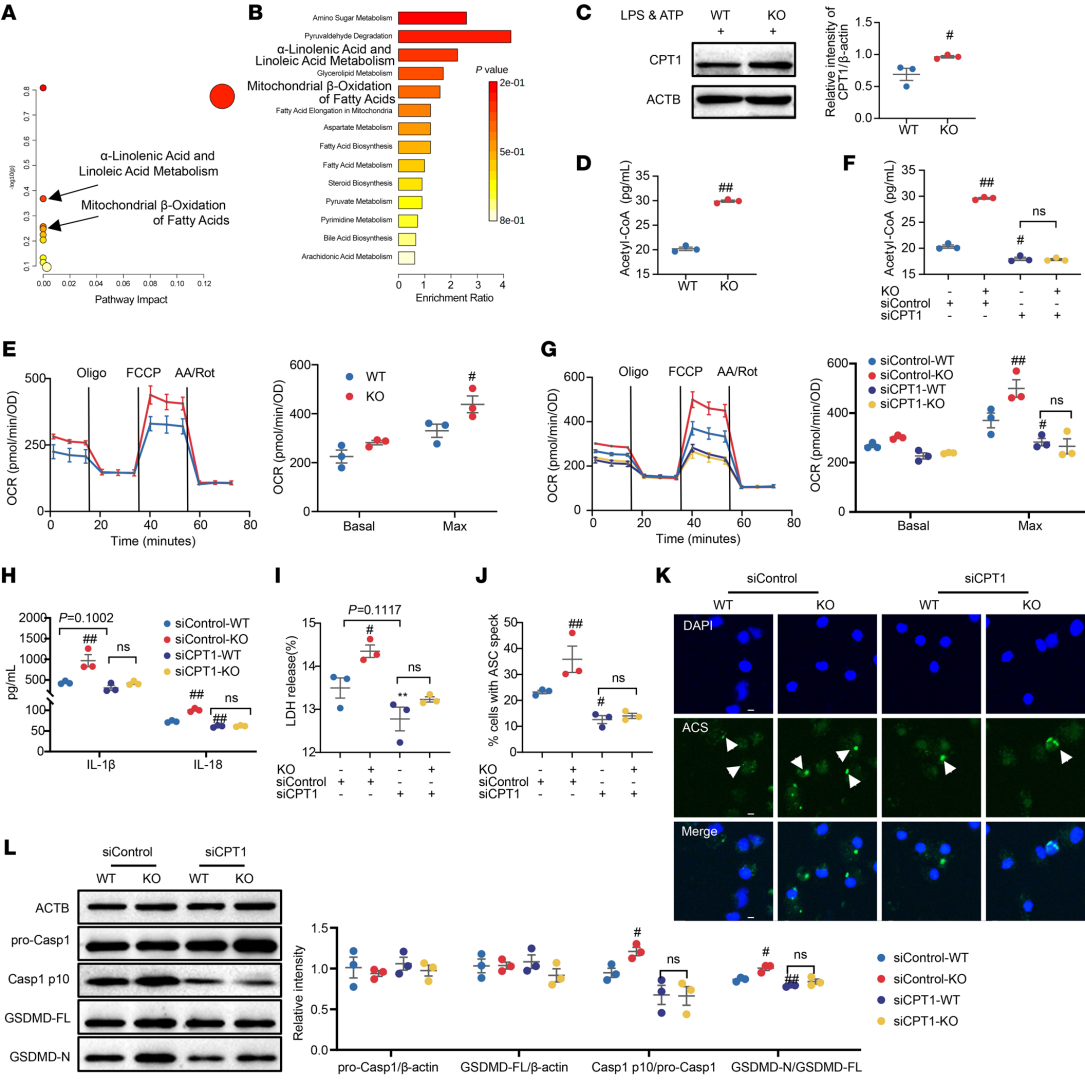
Effect of CPT1 knockdown on ERβ deficiency–driven macrophage pyroptosis. (**A**–**E**) Primary mouse PMs were treated with LPS (2 μg/mL) for 3 hours, followed by ATP (5 mM) treatment for 1 hour. (**A** and **B**) Pathway analysis between pyroptosis group and ERβ activation group. *n* = 6. The two pathways displayed in enlarged font, α-linolenic acid and linoleic acid metabolism, and mitochondrial β-oxidation of fatty acids, are key findings in our study. (**C**) The protein expression of CPT1 was analyzed by Western blot. *n* = 3. (**D**) The acetyl-CoA levels of PMs were detected by using commercial kit. *n* = 3. (**E**) Oxygen consumption rate of PMs was monitored by Seahorse XFe96 analyzer. *n* = 3. (**F**–**L**) The primary mouse PMs, transfected with siControl or siCPT1, were treated with LPS (2 μg/mL) for 3 hours, followed by ATP (5 mM) treatment for 1 hour. *n* = 3. (**F**) The acetyl-CoA levels in PMs were detected by using commercial kit. (**G**) Oxygen consumption rate of PMs was monitored by Seahorse XFe96 analyzer. (**H**) IL-1β and IL-18 levels and (**I**) LDH release were measured in the supernatants of PMs. (**J** and **K**) The ASC oligomerization in PMs was detected by immunofluorescence assay. (**L**) The expression of pro-Casp1, Casp1 p10, GSDMD-FL, and GSDMD-N in PMs was detected by Western blot. Unpaired Student’s *t* test was performed in **C**–**E**. Two-way ANOVA was employed in **F**–**J** and **L**. Data are expressed as the means ± SEM. *n* = 3. ^#^*P* < 0.05 and ^##^*P* < 0.01 versus WT group or siControl + WT group. Scale bars: 5 μm.

As energy metabolism plays an important role in the regulation of pyroptosis (31), we investigated the effect of ERβ on energy metabolism pathways under conditions of pyroptosis. UHPLC-Q-TOF-MS–based untargeted metabolomics were used to examine the metabolite profile of mouse PMs to identify the major metabolic pathways affected by ERβ activation (Supplemental Figure 5, A–D). Metabolomics results showed that, under pyroptosis conditions, the major affected metabolic pathways (top 5) include α-linolenic acid and linoleic acid metabolism, vitamin B6 metabolism, plasmalogen synthesis, arachidonic acid metabolism, and mitochondrial β-oxidation of fatty acids (Supplemental Figure 5, E and F). Compared with the pyroptosis group, the major metabolic pathways influenced by ERβ activation (top 5) include amino sugar metabolism, pyruvaldehyde degradation, α-linolenic acid and linoleic acid metabolism, glycerolipid metabolism, and mitochondrial β-oxidation of fatty acids (Figure 4, A and B). Notably, both α-linolenic acid and linoleic acid metabolism as well as mitochondrial β-oxidation of fatty acids are changed under pyroptosis conditions under both pyroptosis conditions and ERβ activation interventions. Linoleic acid and α-linolenic acid are more readily subjected to β-oxidation in mitochondria and peroxisomes compared with other long-chain fatty acids, such as oleic acid and palmitic acid (32). The β-oxidation of long-chain fatty acids is a central pathway in fatty acid oxidation (FAO). This suggests that ERβ deficiency–induced macrophage pyroptosis may occur through the modulation of the FAO pathway. In vitro results showed that ERβ deficiency significantly upregulated the expression of carnitine acyl transferase 1 (CPT1; the key rate-limiting enzyme of FAO), upregulated the intracellular acetyl-CoA level, and increased the basal and maximum oxygen consumption of macrophages under the condition of pyroptosis (Figure 4, C–E). These results suggest that ERβ deficiency significantly enhances macrophage FAO under pyroptosis conditions. Subsequently, we examined the involvement of FAO in the promotion of macrophage pyroptosis by ERβ deficiency. We constructed 3 siRNAs of CPT1, and qPCR results showed that siCPT1-1 had the highest knockdown efficiency, which was used for subsequent experiments (written as siCPT1) (Supplemental Figure 6B and Figure 4, F and G). In vitro results indicated that under pyroptotic conditions, the increased proportion of macrophage death induced by ERβ deficiency was significantly suppressed by CPT1A knockdown (Supplemental Figure 6C). Furthermore, the enhancing effects of ERβ deficiency on pyroptosis-mediated IL-1β and IL-18 secretion, LDH release, and apoptosis-associated speck-like protein containing a CARD (ASC) oligomerization and the elevated expression of pyroptosis-related proteins were all markedly attenuated by siCPT1 (Figure 4, H–L). Collectively, these data suggest that ERβ deficiency promotes macrophage pyroptosis by augmenting FAO.

To investigate the involvement of CPT1-mediated FAO in the exacerbation of sepsis by ERβ deficiency, mice were intraperitoneally injected with the CPT1 inhibitor etomoxir or vehicle 1 hour before establishing the CLP model (33). The results showed that the exacerbation of disease symptoms in CLP-induced septic mice caused by ERβ deficiency was significantly inhibited by etomoxir treatment (Supplemental Figure 7). This result suggests that ERβ deficiency exacerbates sepsis in mice by enhancing CPT1-mediated FAO.

### ERβ deficiency promotes the acetylation of pyroptosis-related proteins by enhancing FAO in PMs.

**Figure 5:**
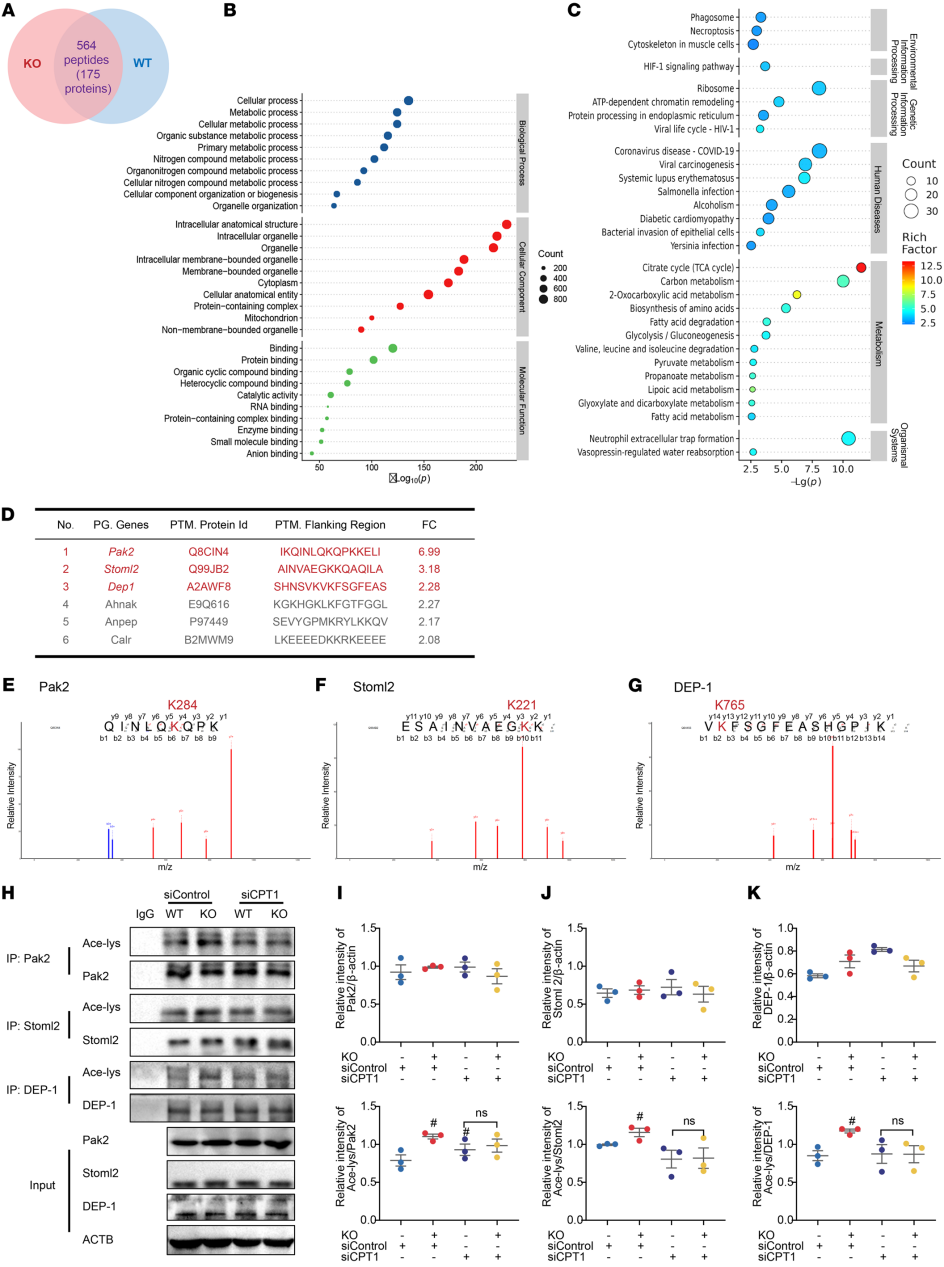
Effect of ERβ deficiency on protein acetylation modification in macrophages and the involvement of CPT1. (**A**–**G**) Primary mouse PMs were treated with LPS (2 μg/mL) for 3 hours, followed by ATP (5 mM) treatment for 1 hour. (**A**) The number of proteins with upregulated acetylation modification levels and their corresponding modification sites. (**B**) GO term enrichment analysis of key targets. (**C**) KEGG pathway enrichment analysis of key targets (top 30 were listed). (**D**) Table (top 6) of the proteins corresponding to the differential sites in the KO versus WT groups related to pyroptosis. (**E**–**G**) The mass spectrum of Pak2, Stoml2, and DEP-1 acetylation sites in KO mice compared with WT mice. (**H**–**K**) The primary mouse PMs, transfected with siControl or siCPT1, were treated with LPS (2 μg/mL) for 3 hours, followed by ATP (5 mM) treatment for 1 hour. *n* = 3. The acetylation of lysine of Pak2, Stoml2, and DEP-1 in PMs was measured by using co-IP. Two-way ANOVA was employed in **I**–**K**. Data are expressed as the means ± SEM. *n* = 3. ^#^*P* < 0.05 versus siControl + WT group.

Protein acetylation plays a pivotal role in regulating protein structure and function, with acetyl-CoA serving as a key donor for this modification (34). ERβ deficiency significantly elevated acetyl-CoA levels in macrophages during pyroptosis (Figure 4D). However, it remains unclear whether protein acetylation is upregulated in this context and whether this modification contributes to the enhanced macrophage pyroptosis induced by ERβ deficiency. To address this, we performed quantitative acetylome analysis of primary mouse PMs under pyroptosis conditions. We identified the upregulation of 564 acetylated peptides of 175 different proteins after ERβ knockout (Figure 5A). Among these proteins, p21 (RAC1) activated kinase 2 (Pak2; K284), stomatin-like protein 2 (Stoml2; K221), and density-enhanced phosphatase-1 (DEP-1; K765) were closely related to macrophage pyroptosis (Figure 5, B–G). In vitro results showed that ERβ deficiency significantly upregulated the acetylation levels of Pak2, Stoml2, and DEP-1 compared with the WT group (Supplemental Figure 8). Combined with siCPT1, ERβ deficiency–induced upregulation of Pak2, Stoml2, and DEP-1 acetylation was markedly inhibited, suggesting ERβ deficiency promotes the acetylation of Pak2, Stoml2, and DEP-1 by enhancing FAO in macrophages (Figure 5, H–K).

### ERβ deficiency drives macrophage pyroptosis by promoting Stoml2 K221 acetylation.

**Figure 6:**
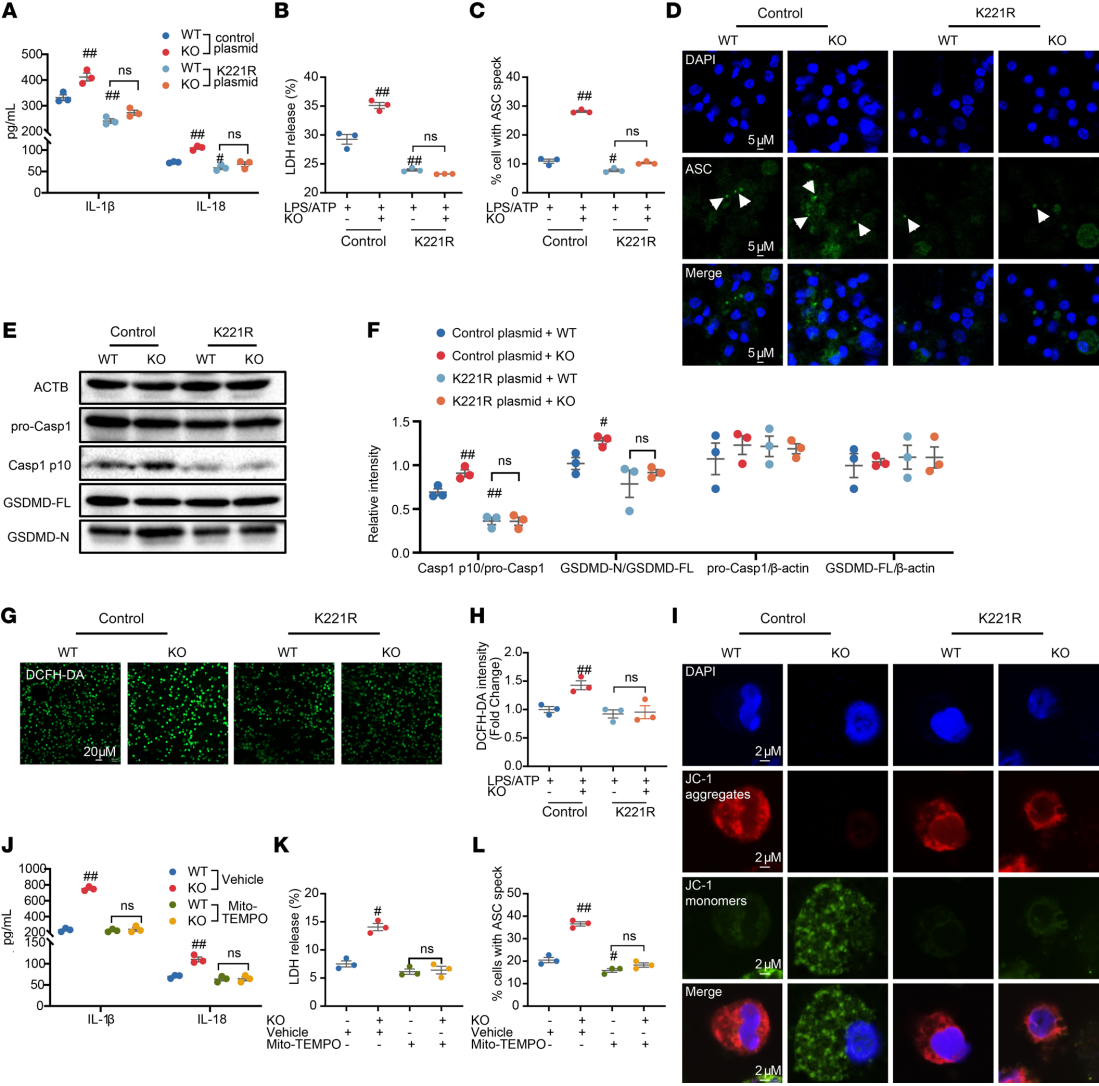
Effect of mutations in the acetylated lysine site of Stoml2 on promotion of ERβ deficiency against macrophage pyroptosis. (**A**–**H**) The primary mouse PMs, transfected with either control plasmid or K221R plasmid, were treated with LPS (2 μg/mL) for 3 hours, followed by ATP (5 mM) treatment for 1 hour. *n* = 3. (**A**) IL-1β and IL-18 levels and (**B**) LDH release were measured in the supernatants of PMs. (**C** and **D**) The ASC oligomerization in PMs was measured by using immunofluorescence assay (scale bars: 5 μm). (**E** and **F**) The protein expression of Casp1 p10, pro-Casp1, GSDMD-FL, and GSDMD-N of PMs was analyzed by Western blot. (**G** and **H**) ROS level in PMs was measured by using immunofluorescence assay (scale bars: 20 μm). (**I**) JC-1 signals in PMs were measured by using immunofluorescence assay (scale bars: 2 μm). (**J**–**L**) The primary mouse PMs, pretreated with mitochondria specific ROS inhibitor Mito-TEMPO (50 nM, HY-112879, MCE) or vehicle for 24 hours, were treated with LPS (2 μg/mL) for 3 hours, followed by ATP (5 mM) treatment for 1 hour. *n* = 3. (**J**) IL-1β and IL-18 levels and (**K**) LDH levels in the supernatants of PMs were measured. (**L**) The ASC oligomerization in PMs was measured by using immunofluorescence assay. Two-way ANOVA was employed in **A**–**C**, **F**, **H**, and **J**–**L**. Data are presented as mean ± SEM. *n* = 3. ^#^*P* < 0.05 and ^##^*P* < 0.01 versus control plasmid + WT group.

To further screen the effector proteins and their corresponding sites involved in ERβ deficiency–promoted macrophage pyroptosis, point mutant plasmids were constructed by mutating the K284 residue of Pak2, the K221 residue of Stoml2, and the K765 residue of DEP-1 to arginine, thereby mimicking protein deacetylation in primary mouse PMs. The effect of ERβ deficiency on the levels of IL-1β and IL-18, LDH release, and ASC oligomerization and the expression of Casp1 p10, pro-Casp1, GSDMD-FL, and GSDMD-N following the acetylated lysine site mutation of the target proteins was investigated using ELISA, immunofluorescence, and Western blot. Mutating the K284 residue of Pak2 and the K765 residue of DEP-1 to arginine did not alter the impact of ERβ deficiency on macrophage pyroptosis (Supplemental Figure 9). Mutation of the K221 residue in Stoml2 significantly diminished the impact of ERβ deficiency on macrophage pyroptosis, indicating that ERβ deficiency drives macrophage pyroptosis by promoting acetylation at the K221 residue of Stoml2 (Figure 6, A–F).

Stoml2, a member of the stomatin superfamily, is mainly located in the inner mitochondrial membrane and can regulate mitochondrial function through a variety of pathways (35). It has been reported that Stoml2 overexpression substantially upregulates mitochondrial membrane potential (MMP) (36), while Stoml2 knockout markedly increases mitochondrial reactive oxygen species (ROS) levels and intracellular ROS levels and enhances mitochondrial dysfunction in macrophages (37). Mitochondrial dysfunction and the release of mitochondrial ROS into the cytoplasm are the key upstream events during macrophage pyroptosis (38). To explore the mechanism by which ERβ deficiency upregulates Stoml2 acetylation to promote pyroptosis, we investigated the effect of ERβ deficiency on intracellular ROS levels and MMP in macrophages. Immunofluorescence results showed that ERβ deficiency significantly upregulated intracellular ROS levels and downregulated MMP to exacerbate mitochondrial dysfunction in macrophages (Figure 6, G–I). The Stoml2 K221 mutation significantly reduced the aggravated effect of ERβ deficiency on mitochondrial dysfunction (Figure 6, G–I). Finally, we combined the use of the mitochondria specific ROS inhibitor Mito-TEMPO to suppress mitochondrial ROS production (28). The results showed that the exacerbating effect of ERβ deficiency on macrophage pyroptosis was significantly inhibited by Mito-TEMPO treatment (Figure 6, J–L, and Supplemental Figure 6, D and E). These findings suggest that ERβ deficiency drives macrophage pyroptosis by promoting acetylation at the K221 site of Stoml2, which in turn induces mitochondrial dysfunction.

### ERβ deficiency worsens sepsis outcomes by promoting Stoml2 K221 acetylation.

**Figure 7:**
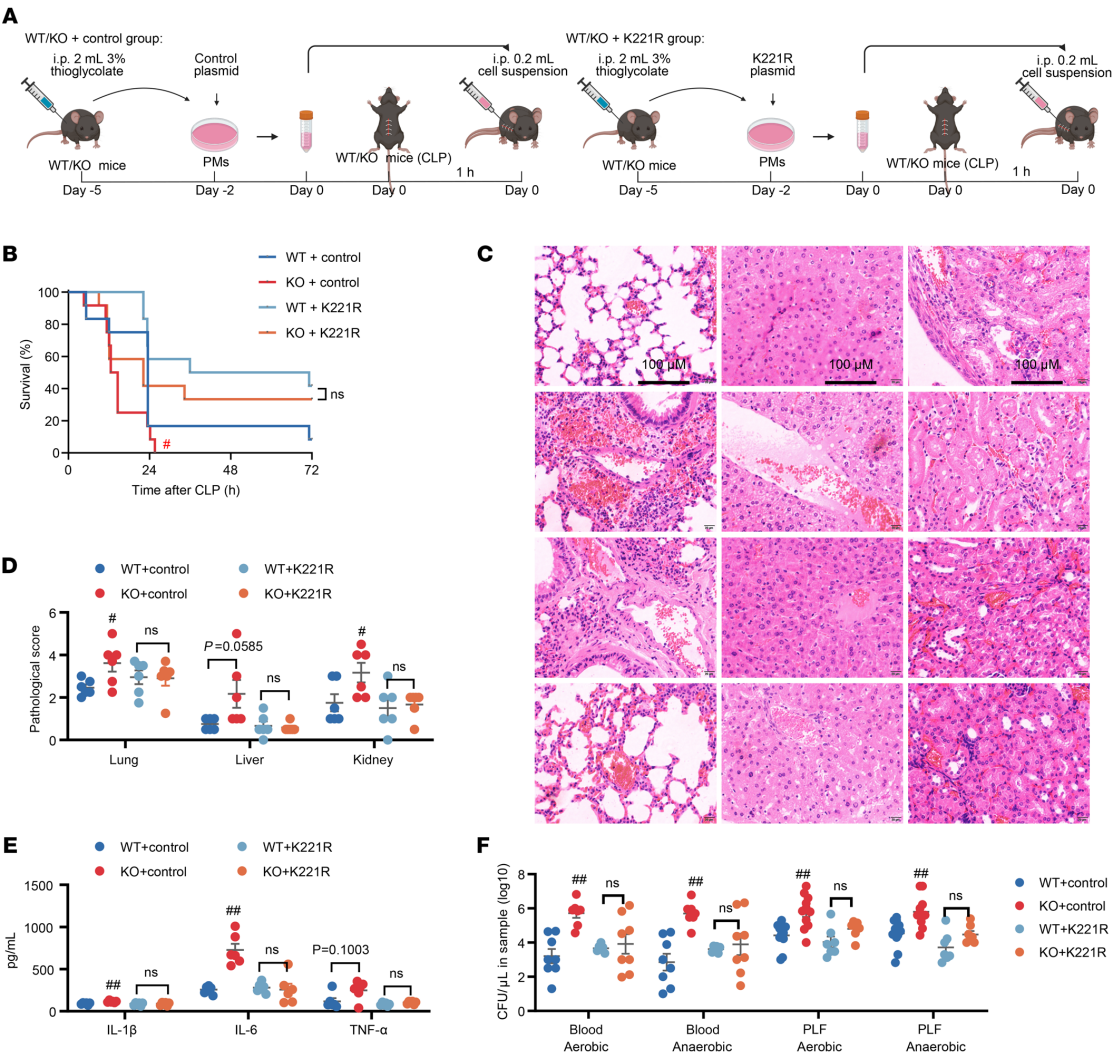
Effect of mutations in the acetylated lysine site of Stoml2 on the exacerbation of sepsis outcomes induced by CLP in the context of ERβ deficiency. (**A**) The study protocol is shown. Adoptive transfer of PMs with the Stoml2 K221 point mutation into corresponding CLP-induced sepsis mice. (**B**) The survival curve of septic mice. *n* = 12. (**C** and **D**) H&E staining and histopathological scores of the lung, liver, and kidney from septic mice. *n* = 6. (**E**) Quantification of cytokines in the sera from septic mice. *n* = 6. (**F**) Quantification of bacterial colonies in the blood and PLF from septic mice. *n* = 6–11. Log-rank (Mantel-Cox) test was adopted to compare the significance in **B**. Two-way ANOVA was employed in **D**–**F**. Data are presented as mean ± SEM. ^#^*P* < 0.05 and ^##^*P* < 0.01 versus WT + control group. Scale bar, 100 μm.

Based on the aforementioned findings, we hypothesized that adoptive transfer of PMs with the Stoml2 K221 point mutation can significantly mitigate the increased susceptibility of mice to sepsis induced by ERβ deficiency. To verify this hypothesis, we established a sepsis model in mice induced by CLP and performed adoptive transfer of the corresponding PMs into the different groups of mice (Figure 7A). In vivo results showed that, compared with the WT + control group, the KO + control group exhibited more severe symptoms of the disease, as shown by higher mortality; aggravated multiorgan damage; increased serum concentrations of IL-1β, IL-6, and TNF-α; and elevated viable bacteria in blood and PLF (Figure 7, B–F). However, when Stoml2 K221 was mutated to arginine, the aggravating effect of ERβ deficiency on sepsis was completely offset (Figure 7, B–F). Similar results were seen from mice challenged with LPS (Supplemental Figure 10). Taken together, these results suggest that ERβ deficiency increases the susceptibility of mice to sepsis by promoting Stoml2 K221 acetylation.

## Discussion

Here, we report 4 important findings on the role of ERβ in sepsis: (i) in sepsis patients, the expression levels of ERβ are significantly decreased and are negatively correlated with disease severity; (ii) ERβ deficiency drives macrophage pyroptosis to impair bacterial clearance, ultimately increasing the body’s susceptibility to sepsis; (iii) ERβ deficiency drives macrophage pyroptosis by enhancing the intracellular FAO pathway; and (iv) promotion of Stoml2 K221 acetylation is critical for ERβ deficiency to drive macrophage pyroptosis and consequently increase the susceptibility to sepsis in mice. Overall, these findings highlight ERβ deficiency as a potential genetic factor in sepsis susceptibility. ERβ has the potential to serve as a predictive biomarker for sepsis symptoms, and interfering with Stoml2 acetylation may represent a potential host-directed therapy to restore immune homeostasis under sepsis status.

Sepsis, a multifactorial disease, often originates from the lungs, urinary tract, and abdomen (24). It is more prevalent in males (incidence rate, male/female = 1.37:1), with male patients with sepsis exhibiting higher hospitalization mortality and recurrence rates (39.3%; 22.3%) compared with female patients (33.7%; 19.4%) (12). ERβ expression in both the lungs and urinary tract also exhibits sex-based differences (13–15). Clinical studies have shown that the proportion of ERβ-positive primary lung tumor tissue in women is higher (44%) compared with men (31%), while the proportion of ERβ-positive urinary tract epithelium in healthy women (35%) is markedly higher than in men (undetectable) (16, 17). This suggests that the sex bias in the onset and progression of sepsis may be associated with the expression level of ERβ in the body. Furthermore, multiple clinical studies indicate that patients with reduced ERβ expression at the lesion site have an increased risk of bloodstream infections and are more susceptible to sepsis-related organ damage (18, 19). Compared with healthy people, there is a trend of decreased ERβ expression in the hypothalamic supraoptic nucleus of patients with depression, and severe depressive symptoms are associated with an increased risk of bloodstream infections (18, 19). Based on the foregoing, the expression of ERβ may be linked to susceptibility to sepsis. In this study, we collected whole blood samples from 26 patients and 30 healthy controls. qPCR assay showed that the mRNA expression of ERβ and its target genes in the whole blood of sepsis was markedly lower than that of healthy controls on the day of admission, and the mRNA expression of ERβ and its target genes was negatively correlated with the severity of sepsis. ERs include ERα and GPER in addition to ERβ, and we also examined the expression of ERα and GPER in whole blood samples from patients and healthy controls. The results showed that there was no significant difference in *ESR1* and *GPER1* expression between whole blood samples from patients and controls. This suggests that ERβ expression may be closely related to the occurrence and development of sepsis. Subsequently, we also demonstrated that ERβ deficiency increased the susceptibility to sepsis and exacerbated disease symptoms in CLP- and LPS-induced mouse sepsis.

The mortality of sepsis is closely linked to the failure to eliminate invading pathogens from the body (24, 25). In a postmortem study conducted on 235 surgical intensive care unit (ICU) patients admitted with sepsis, approximately 80% of patients had unresolved septic foci at the time of death (39). Phagocytic cells play a crucial role in defending the body against bacterial pathogens (25). Phagocytic cells detect bacteria through pattern recognition receptors, classify them, and subsequently initiate corresponding signaling cascades to trigger defense mechanisms within phagocytes and mobilize other immune cells to act (25). The proportion of phagocytes is closely related to the progression of sepsis and its associated complications (40). In the PLF of mice with peritonitis induced by yeast glucan A, the proportion of macrophages was significantly downregulated but gradually returned to normal proportions as the disease progressed (40). A reduction in the number of phagocytic cells exacerbates the symptoms of sepsis (27, 41, 42). Administration of clodronate or neutralizing antibodies to selectively deplete macrophages or neutrophils in mice results in an increased mortality rate in CLP-induced septic mice (27). Macrophage depletion exacerbates CLP-induced septic acute kidney injury in mice, as well as symptoms in both LPS-induced septic mice and cecal slurry shock mice (41, 42). Compared with nonpregnant mice, pregnant mice had a downregulated proportion of PMs and increased susceptibility to CLP- and LPS-induced sepsis in mice (27). In contrast, the upregulation of phagocyte proportions can ameliorate sepsis and its associated complications in mice (43–45). Administering proliferative tissue-resident macrophages into the pericardium of mice can enhance cardiac function and prevent cardiomyopathy induced by CLP-induced sepsis (43). In a mouse model of peritonitis induced by zymosan A, phagocytosis can promote macrophage proliferation, ultimately improving tissue damage in the mice (44). In an experimental model of sepsis-associated acute respiratory distress syndrome (ARDS) induced by LPS aerosol inhalation, administration of resolvin D1 to ARDS mice promotes the proliferation of TRMs in the lung, enhances their phagocytic capacity, and ameliorates the disease symptoms in the mice (45). Hence, it is essential to investigate the impact of ERβ deficiency on the proportion of phagocytes during sepsis. CBC data revealed that neutrophil proportions remained unchanged in male septic patients with differential ERβ expression, and those with low ERβ expression exhibited a significant reduction in monocyte/macrophage proportions compared with patients with high ERβ expression during sepsis (Figure 3A). In vivo, ERβ deficiency reduced macrophage proportions in PLF of septic mice without affecting neutrophil proportions. Subsequently, we further demonstrated that ERβ deficiency did not interfere with monocyte differentiation, macrophage recruitment, and the proportion of TRMs but significantly promoted macrophage death. To better understand the predominant form of cell death contributing to the reduction in macrophage numbers during sepsis, mice subjected to CLP-induced sepsis were administered specific inhibitors targeting various cell death pathways, including VX765, ferrostatin-1, necrostatin-1, and Z-DEVD-FMK. Our findings revealed an increase in PMs in septic mice treated with the caspase-1 inhibitor VX765. The above results suggest that in the septic state, the ERβ deficiency leads to a downregulation of the proportion of macrophages primarily by driving pyroptosis.

Although we observed that systemic ERβ-knockout mice exhibited increased susceptibility, the current experimental design primarily focused on the intrinsic mechanisms of macrophages and did not fully elucidate the specific role of ERβ in adaptive immune cells (such as T and B cells). Notably, it is reported that the ERβ-specific agonist diarylpropionitrile can concentration-dependently inhibit IFN-γ production in activated splenic lymphocytes, and IFN-γ has been confirmed to significantly induce pyroptosis in various cells (46–48). This implies that ERβ deficiency may lead to T cell dysfunction and increased IFN-γ secretion and subsequently exacerbate macrophage pyroptosis through paracrine effects, forming a cascade-amplified inflammatory loop involving “adaptive immunity-innate immunity.” Although this hypothesis was not directly validated in the present study, our study provides important insights for understanding the systemic regulatory role of ERβ within the immune network and highlights valuable directions for future research: utilizing conditional gene knockout mouse models (e.g., T cell–specific ERβ knockout) to dissect the specific functions of ERβ signaling in different immune cell subsets; investigating the interplay between cytokines, such as IFN-γ, and the Stoml2 acetylation pathway; and developing combined intervention strategies targeting the ERβ/IFN-γ axis, which may offer novel therapeutic targets for immunomodulatory treatments in sepsis.

Numerous studies have shown that energy metabolism plays a crucial role in the process of pyroptosis (49–51). An intermediate product of the tricarboxylic acid cycle, succinate, mediates gasdermin D succination, which prevents its interaction with caspases, thereby limiting its processing, oligomerization, and capacity to induce pyroptosis (49). Similarly, another key intermediate of the tricarboxylic acid cycle, α-ketoglutarate, induces pyroptosis through caspase-8–mediated cleavage of gasdermin C (50). Inhibition of fatty acid synthetase suppresses NLRP3 inflammasome–dependent endothelial cell pyroptosis via activation of the Nrf2/HO-1 pathway (51). Nevertheless, it remains uncertain whether the contribution of ERβ deficiency to macrophage pyroptosis is mediated through the regulation of energy metabolism. We used untargeted metabolomics to investigate the changes of metabolites in macrophages under the pyroptosis condition. The results demonstrated that ERβ activation modulated the levels of various metabolites in macrophages undergoing pyroptosis. Pathway enrichment analysis further revealed that ERβ activation exerted a regulatory influence on FAO. In vitro results showed that ERβ deficiency enhanced macrophage FAO as indicated by increased CPT1 expression, upregulated acetyl-CoA levels, and elevated basal and maximal macrophage oxygen consumption. Subsequently, the FAO-dependent role of ERβ deficiency in promoting macrophage pyroptosis was demonstrated by a combined use with siCPT1. These results suggest that ERβ deficiency drives macrophage pyroptosis by enhancing FAO.

Protein translational modifications (PTMs) refer to the covalent attachment of functional groups to synthesized proteins, the proteolytic cleavage of regulatory subunits, or the degradation of the entire protein, all of which serve to adjust or alter its properties and functions (52). Fundamental research defining the mechanisms whereby PTMs of proteins regulate diseases will open new avenues for therapeutic intervention (52). Identifying the principal posttranslationally modified proteins and their corresponding sites is essential for the development of drugs targeting cellular pyroptosis. Common PTMs, such as methylation, acetylation, and phosphorylation, are pivotal in regulating protein structure, function, stability, and localization within the cell (53–55). Notably, lysine acetylation at specific sites is an evolutionarily conserved modification, present in both prokaryotes and eukaryotes. Based on quantitative acetylome analysis and site-directed mutagenesis, we identified that under pyroptosis conditions, the primary target protein affected by ERβ deficiency is Stoml2, with the corresponding site being K221. Additionally, in vivo experiments further clarified the involvement of acetylation at the Stoml2 K221 site in the exacerbation of disease symptoms in septic mice due to ERβ deficiency.

Stoml2 is a mitochondrial inner membrane protein and a crucial regulator of mitochondrial function, including changes in MMP, ATP production, assembly of respiratory chain complexes, and mitochondrial fission and fusion (35). Stoml2 can form hetero-oligomers with the mitochondrial fusion protein 2, thereby upregulating MMP (36). Mitochondrial calcium overload is one early event in cell apoptosis. Stoml2 inhibits cisplatin-induced apoptosis in cervical cancer cells by stabilizing mitochondrial calcium levels and upregulating MMP (56). Mitochondrial dysfunction is often accompanied by an increase in mitochondrial ROS production and the release of mitochondrial ROS into the cytoplasm, serving as a crucial upstream mechanism in pyroptosis. We found that ERβ deficiency significantly elevated ROS levels in macrophages and downregulated the MMP. The mutation at the K221 site of Stoml2 completely negated the effects of ERβ deficiency, suggesting that ERβ deficiency mediates mitochondrial dysfunction and promotes macrophage pyroptosis by enhancing the acetylation of Stoml2 at K221.

In conclusion, our study indicates that decreased ERβ expression is well correlated with sepsis severity. The role of ERβ deficiency in increasing the body’s susceptibility to sepsis is closely linked to triggering of macrophage pyroptosis, which impairs bacterial clearance. ERβ deficiency drives macrophage pyroptosis by promoting the acetylation of Stoml2. Adoptive transfer of PMs with the Stoml2 K221 point mutation significantly reversed the above effects of ERβ deficiency. ERβ deficiency serves as a potential genetic factor in sepsis susceptibility and could represent an attractive biomarker for the early identification and prevention of sepsis. These findings lay the foundation for future studies guiding the development of personalized ERβ-based immune therapies for sepsis.

## Methods

### Sex as a biological variable.

For clinical samples, both sexes were involved. Our study examined male and female animals, and similar findings are reported for both sexes.

### Human samples.

Patients were diagnosed with sepsis according to the criteria outlined in the Third International Consensus Definition (Sepsis-3) (2). The following exclusion criteria were applied: individuals under 18 years of age; those with preexisting immunosuppression; transplant recipients, patients diagnosed with decompensated cirrhosis (Child-Pugh class B or C), hematological disorders, and conditions requiring hemodialysis such as chronic renal failure; and those with a history of anticoagulant therapy in the 4 weeks prior to admission. A total of 26 patients, admitted to the ICU of the Third Affiliated Hospital of Zhejiang Chinese Medical University between April and October 2024, met these eligibility requirements (43–94 years, 18 male, 8 female, *n* = 26). Blood samples were collected between days 1 and 2 following the diagnosis of sepsis in these patients upon ICU admission. Whole blood was promptly drawn (within 6 hours) and subsequently stored at –80°C once all patients fulfilled the criteria for septic shock, to minimize any potential therapeutic bias. Additionally, blood samples were collected from 30 healthy controls at the same hospital during the same period (ages 14–88 years, 12 male, 18 female, *n* = 30). Patient data, including 28-day mortality, were documented (see Supplemental Table 1).

### Mice.

Eight-week-old *Esr2*^+/–^ mice were procured from the Shanghai Model Organisms Center. The mice were housed in a controlled environment with free access to food and water under a 12-hour light/12-hour dark cycle at a temperature of 23°C ± 2°C. To generate ERβ knockout (KO) mice, a controlled breeding protocol was established utilizing *Esr2*^+/–^ mice of both sexes, followed by genetic testing of their progeny using samples collected from their tail tissues (Supplemental Figure 6A). WT littermates served as comparative controls in this study.

### Mouse sepsis models by CLP and LPS.

CLP was conducted following previously established protocols (27). In brief, C57BL/6 mice were anesthetized deeply with isoflurane, and a 1–2 cm midline laparotomy was performed under sterile conditions to expose the cecum. The distal portion of the cecum was ligated, and a single perforation was made using an 18 G needle, allowing a small amount of fecal material to be extruded. The peritoneum was then closed, and the mice were resuscitated with 1 mL of sterile saline injected subcutaneously. To induce endotoxemia, mice were intraperitoneally injected with LPS at a dose of 20 mg/kg. The humane euthanasia criteria for septic mice, based on prior studies, included 1) body temperature < 30°C, 2) weight loss exceeding 10% of baseline body weight, 3) huddling behavior (inability to eat or lack of movement when manually stimulated), and 4) absence of grooming with ruffled fur. Mice exhibiting 3 or more of these symptoms were euthanized (27).

### Histopathological analysis.

Livers, lungs, and kidneys from septic mice were harvested and preserved in 4% paraformaldehyde for 72 hours. After embedding in paraffin, tissue samples were sectioned into 5 μm thick slices and stained with H&E. Lung injury severity was evaluated using a scoring system from 0 to 4, based on several parameters, including congestion of the pulmonary alveolar walls, edema, inflammatory cell infiltration, emphysema, and perivascular inflammation (27). The severity of liver injury was scored 0 to 4 for each parameter, including hepatocellular degeneration and necrosis; vascular and hepatic sinusoidal congestion and inflammation; and subcapsular edema and inflammatory cell infiltration (27). The severity of kidney injury was scored 0 to 4 for each parameter, including glomerular capsule exudation, tubular epithelial degeneration and cell apoptosis, and tubular interstitial congestion (27). A pathologist, who was unaware of the treatment groups, assessed and scored the histological changes based on predetermined criteria.

### Bacterial load assay.

To assess the bacterial load in both blood and PLF from septic mice, samples were serially diluted 10-fold in sterile PBS. Subsequently, 10 μL from each dilution was plated onto blood agar plates and incubated at 37°C for 24 hours under either aerobic or anaerobic conditions.

### Flow cytometry.

To analyze macrophages and neutrophils in the PLF of mice, the samples were incubated with APC-conjugated anti-mouse Ly6G antibody, PE-conjugated anti-mouse F4/80 antibody, and Alexa Fluor 488–conjugated anti-mouse/human CD11b antibody at 4°C for 1 hour in the dark. For a more detailed assessment of macrophage populations, the peritoneal cavity was lavaged with 5 mL of cold PBS, and 1 mL of the lavage fluid was stained with PE-conjugated anti-mouse F4/80 and FITC-conjugated anti-CD11b antibodies. Flow cytometry was employed to quantify macrophages in PLF obtained from septic mice. To evaluate death of PMs, samples were stained with PE anti-F4/80 or FITC-conjugated anti-mouse F4/80 antibody, FITC anti-CD11b antibodies or bv421-conjugated anti-mouse CD11b antibody, and the Annexin V-APC/7-AAD apoptosis kit at 4°C for 1 hour in the dark. Cell death was identified by the F4/80^+^CD11b^+^annexin V^+^7-AAD^+^ population.

To analyze the proportion of TRMs in the PLF of mice, the samples were incubated with bv421-conjugated anti-mouse CD11b antibody, FITC-conjugated anti-mouse F4/80 antibody, PerCP/Cy5.5-conjugated anti-mouse MHC-II antibody, PE-conjugated anti-mouse Ly6G antibody, PE-conjugated anti-mouse CD19 antibody, and PE-conjugated anti-mouse CD90.2 antibody at 4°C for 1 hour in the dark. Flow cytometry was employed to quantify the proportion of TRMs in PLF obtained from septic mice.

To analyze the proportion of M1 and M2 macrophages in the PLF of mice, the samples were incubated with bv421-conjugated anti-mouse CD11b antibody, FITC-conjugated anti-mouse F4/80 antibody, APC-conjugated anti-mouse CD206 antibody, and PE-conjugated anti-mouse CD86 antibody at 4°C for 1 hour in the dark. Flow cytometry was employed to quantify the proportion of M1 and M2 macrophages in PLF obtained from septic mice. Data analysis was performed using FlowJo software, and the flow cytometry gating strategy is provided in Supplemental Figure 11.

### Cell culture.

Primary macrophages derived from murine BM cells and primary PMs were prepared as previously described (57, 58).

### Transient transfection.

siCPT1, control plasmid and K284R of Pak2, control plasmid and K221R of Stoml2, and control plasmid and K765R of DEP-1 were transfected into PMs using the lipo6000 following the manufacturer’s protocol.

### Adoptive transfer of PMs.

PMs were isolated and transfected with the control plasmid and K221R plasmid of Stoml2 as described above. Approximately 2 × 10^6^ PMs/mouse were intraperitoneally injected into recipient mice that had undergone CLP surgery or intraperitoneal injection of LPS 1 hour prior to injection. The study protocol is shown in Figure 7A and Supplemental Figure 10A.

### Biochemical analysis.

The levels of cytokines TNF-α, IL-6, IL-1β, IL-18, and IL-10 were quantitated with corresponding ELISA kits. LDH released from PMs was quantitated by using a LDH cytotoxicity assay kit. The levels of acetyl-CoA in PMs were quantitated by using an acetyl-CoA ELISA kit. The levels of pyruvate in PMs were quantitated by using a pyruvate assay kit. The activity of pyruvate dehydrogenase (PDH) in PMs was measured using a PDH activity assay kit.

### qPCR assay.

qPCR experiments were performed as described previously (58), and the primer sequences used are listed in Supplemental Tables 2 and 3. Additional resource and reagent information is in Supplemental Table 4.

### Western blot analysis.

Cells were lysed using NP-40 buffer containing protease inhibitors. Following a 25-minute incubation on ice, the lysates were centrifuged at 13,800*g* for an additional 30 minutes. The extracted proteins were then separated by SDS-PAGE, transferred to membranes, and blocked with nonfat milk. The membranes were subsequently incubated overnight at 4°C with primary antibodies. The next day, the membranes were treated with secondary antibodies at 37°C for 1 hour, and protein bands were visualized using the Tanon 5200 imaging system.

### Immunofluorescence microscopy.

PMs were seeded onto culture plates and incubated under standard conditions. The cells were fixed using 4% paraformaldehyde for 15 minutes and subsequently permeabilized with 0.5% Triton X-100 for an additional 15 minutes. To reduce nonspecific staining, the cells were blocked with 5% BSA at room temperature for 1 hour. Following blocking, the cells were incubated overnight at 4°C with the appropriate primary antibodies. After washing with PBS, the cells were further treated with fluorescently labeled secondary antibodies and DAPI for nuclear staining. Finally, images were captured using a laser confocal microscope (Carl Zeiss LSM 800).

### Co-immunoprecipitation.

PMs were lysed by incubation on ice for 15 minutes in NP-40 buffer. The lysates were then centrifuged at 13,800*g* for 5 minutes to collect the supernatants. Experiments were performed according to the protein A/G magnetic beads instructions.

### Oxygen consumption rate and glycolysis proton efflux rate analysis.

The oxygen consumption rate was measured with a Seahorse XF96 Bioanalyzer or Seahorse XF24 Bioanalyzer using the Seahorse XF palmitate oxidation stress test kit according to the manufacturer’s instructions (Agilent). The glycolysis proton efflux rate was measured with a Seahorse XF24 Bioanalyzer using the Seahorse XF Glycolytic Rate Assay kit according to the manufacturer’s instructions (Agilent) (49).

### Nontarget metabolomics.

The intracellular metabolic profile was analyzed using an Agilent 1290 UHPLC system coupled with an Agilent 6500 Q-TOF/MS in tandem mode (59). Chromatographic separation was performed on an Agilent Waters ACQUITY T3 column (2.1 mm × 100 mm, 1.8 μm). The mobile phase consisted of 0.1% formic acid in water (A) and 0.1% formic acid in acetonitrile (B). The gradient elution program was as follows: 0 minute, 95% A; 2 minutes, 95% A; 5 minutes, 60% A; 10 minutes, 45% A; 11 minutes, 5% A; 22 minutes, 5% A; 27 minutes, 95% A; 30 minutes, 95% A. Mass spectrometric analysis was conducted on an Agilent Q-TOF 6500, equipped with an Agilent Jet Stream ESI source. The scan range covered 50 to 1,700 *m/z* in both positive and negative ion modes. The following operational parameters were used: nebulizer pressure at 35 psi, sheath gas temperature at 320°C, sheath gas flow at 8 L/min, and capillary voltage set to 4,000 V (+) and 3,500 V (−). Data acquisition was carried out with online automatic calibration using reference ions.

### Quantitative acetylome analysis.

The procedures were performed by Shanghai Bioprofile Technology Co., Ltd. (60). The samples were ground in liquid nitrogen and extracted with approximately 200 μL of SDT lysis buffer. The protein concentration of the extracted proteins was quantified using the BCA protein assay kit. The quantified proteins were digested into peptides using trypsin. Peptides were then acetylated and enriched using the PTMScan Acetyl-lysine motif kit. The peptides were processed for UHPLC-MS/MS analysis. Chromatographic separation was achieved on a Vanquish Neo UHPLC system. Mass spectrometry analysis was performed on an Orbitrap Astral mass spectrometer. The mass spectrometry data were analyzed using Spectronaut (version 18, Biognosys AG) software against the protein database uniprotkb-*Mus musculus* (Mouse) [10090]-87434-20240725.fasta.

### MMP.

The MMP was evaluated using a laser confocal microscope (Carl Zeiss LSM 800), and the JC-1 MMP assay kit was used in accordance with the manufacturer’s guidelines.

### Intracellular ROS level.

The intracellular ROS levels were measured by laser confocal microscope (Carl Zeiss LSM 800) using ROS assay kit according to the manufacturer’s instructions.

### Statistics.

The data are expressed as the mean ± SEM. Statistical analyses were performed using Prism 8.0 software (GraphPad). Pearson’s correlation analysis and the log-rank (Mantel-Cox) test were used for correlation or survival analysis. Two-tailed Student’s *t* test was used to compare the means between 2 independent samples. One-way ANOVA was used to compare the means of the independent samples among multiple groups, and 2-way ANOVA was performed on ERβ knockout and intervention factors using Tukey’s honestly significant difference test. A *P* value of less than 0.05 was considered statistically significant.

### Study approval.

This study was approved by the Ethics Committee of the Third Affiliated Hospital of Zhejiang Chinese Medical University, Hangzhou, China (ZSLL-ZN-2024-023-01), in accordance with the principles of the Declaration of Helsinki. Written informed consent was obtained from the patients or their relatives for this study. All animal experiments were performed in accordance with the ethical guidelines and approved procedures of the Animal Experimentation Ethics Committee of China Pharmaceutical University, Nanjing, China (YSL-202504130).

### Data availability.

All data associated with this study are present in the paper or the supplement. The data reported in this paper have been deposited in the OMIX, China National Center for Bioinformation / Beijing Institute of Genomics, Chinese Academy of Sciences (https://ngdc.cncb.ac.cn/omix/release/OMIX014575). The mass spectrometry proteomics data have been deposited to the ProteomeXchange Consortium (https://www.iprox.cn//page/SCV017.html?query=PXD073393) via the iProX partner repository with the dataset identifier PXD073393. Additional data supporting the study are available in the Supporting Data Values file and can be obtained from the corresponding authors.

## Author contributions

YZ and YD designed the study with input from other authors. YZ performed most experiments and data analysis. GL, Wanyi Zhang, and PG completed the collection of human samples. YG and YH performed some of the mouse experiments. ZW and YX assisted with the metabolomics data analysis and interpretation. LG, JZ, HL, and Wenjie Zhang analyzed MS data. YZ and YD drafted the manuscript. YD and YX take responsibility for the overall content of this study. All authors discussed the results and approved the manuscript. The order of the co–first authors were determined based on their equal contribution to the research. The specific order was agreed upon by authors, considering their respective roles in the research, with no distinction in the significance of their contributions.

## Conflict of interest

The authors have declared that no conflict of interest exists.

## Funding support

- YD by the National Natural Science Foundation of China (No. 82073861).
- YX by the National Natural Science Foundation of China (No. 82174049).

## Supplementary Material

Supplemental data

Unedited blot and gel images

Supporting data values

## Version 1 03/17/2026

In-Press Preview

## Version 2 05/15/2026

Electronic publication
