# Supplementary material for: Estrogen receptor β deficiency increases susceptibility to sepsis through metabolic reprogramming–induced macrophage pyroptosis
Source: J Clin Invest. 2026 Mar 17;136(10):e196636. doi: 10.1172/JCI196636 (PMC13178654; doi:10.1172/JCI196636)
Supplement: Supplemental data [file jci-136-196636-s279.pdf]

Supplementary materials

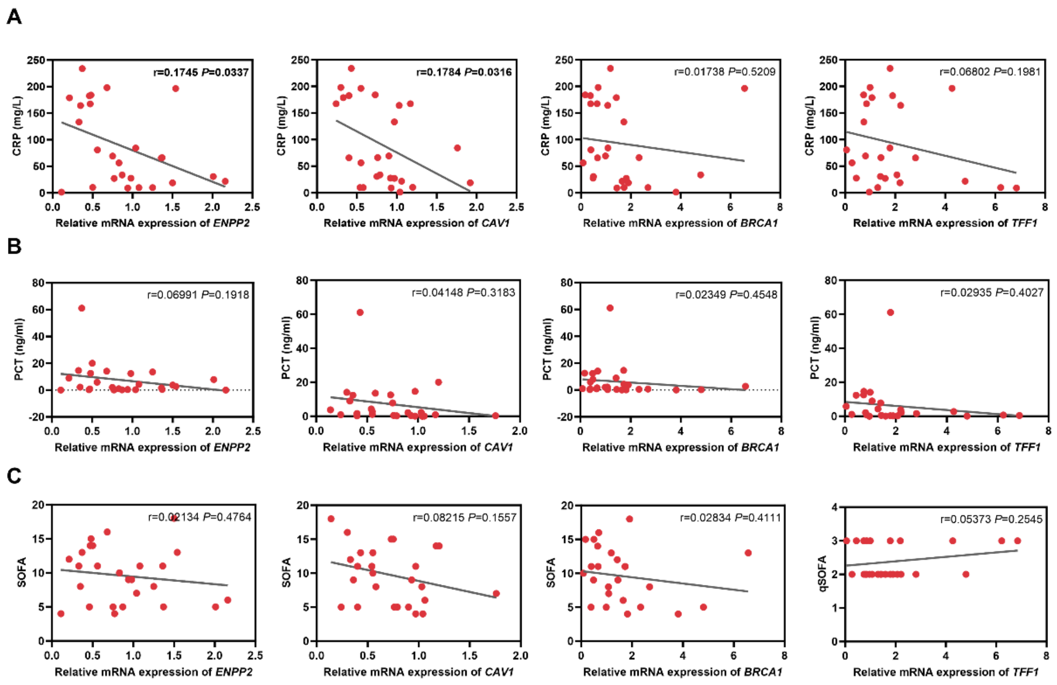

Fig. S1 Correlation analysis of *ENPP2*, *CAV1*, *BRCA1*, *TFF1* expression and disease severity in patients with sepsis. Whole blood samples from patients with sepsis were collected. n = 26. (A) Correlation between the mRNA expression of *ENPP2*, *CAV1*, *BRCA1*, *TFF1* and CRP levels in the blood samples of sepsis patients was analyzed by Pearson correlation analysis. (B) Correlation between the mRNA expression of *ENPP2*, *CAV1*, *BRCA1*, *TFF1* and PCT levels in the blood samples of sepsis patients was analyzed by Pearson correlation analysis. (C) Correlation between the mRNA expression of *ENPP2*, *CAV1*, *BRCA1*, *TFF1* and SOFA of sepsis patients was analyzed by Pearson correlation analysis. Pearson correlation analysis was used in A-C. Data are expressed as mean ± SEM. *r*, Pearson correlation coefficient.

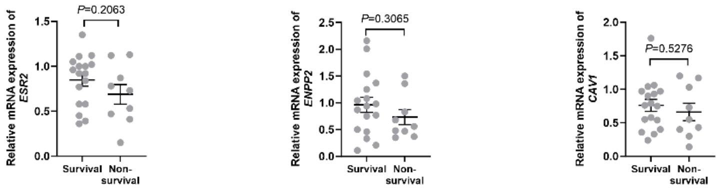

Fig. S2 The mRNA expression of *ESR2*, *ENPP2* and *CAV1* in whole blood samples of patients with sepsis. Sepsis patients were divided into 28 days survivors (n = 17) and non-survivors (n =9), and whole blood samples from patients with sepsis (n = 26) were collected. The mRNA expression of *ESR2*, *ENPP2* and *CAV1* in whole blood samples of survivors and non-survivors was analyzed using qPCR. Unpaired student *t*-test was performed in S2.

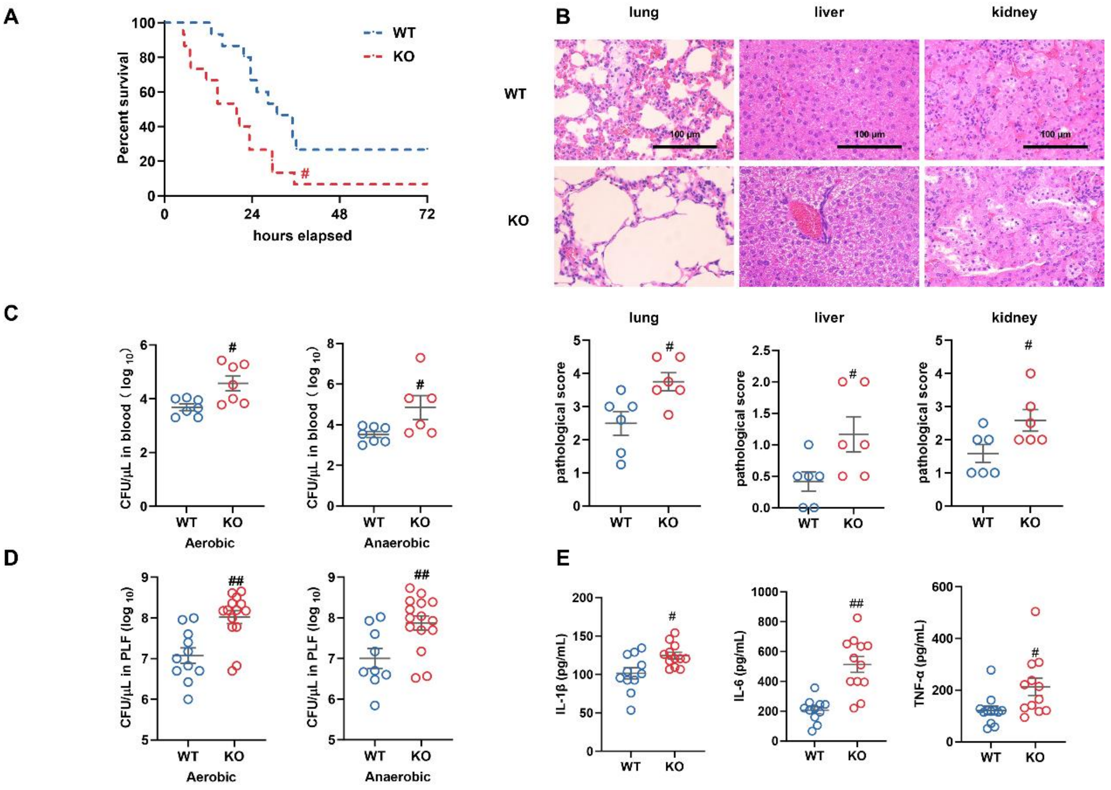

Fig. S3 Effect of ERβ deficiency on disease severity in female septic mice. The female WT and KO mice were subjected to CLP. (A) The survival curve of septic mice,

n = 15. (B) H&E staining and histopathological scores of the lung, liver and kidney from septic mice. n=6. (C and D) Quantification of bacterial colonies in the blood (C) and PLF (D) from septic mice. n = 6-15. (E) Quantification of cytokines in the sera from septic mice. n = 11-12. Long rank (Mantel Cox) test was adopted to compare the significance in A. Unpaired student *t*-test was performed in B-E. Data are expressed as mean ± SEM. $^{\#}P < 0.05$ and $^{\#\#}P < 0.01$ *vs*. WT group. Scale bar, 100 μm.

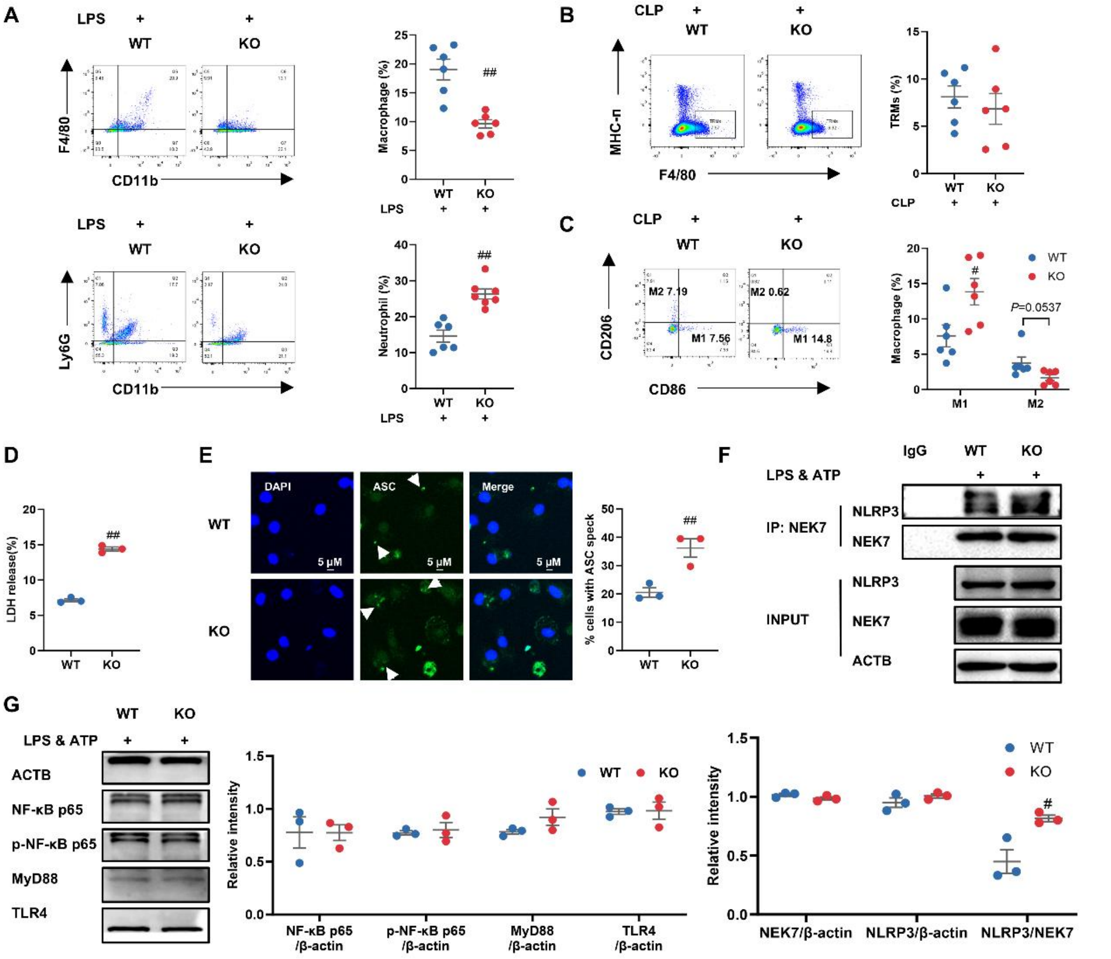

Fig. S4 Effect of ERβ deficiency on the proportion of phagocytic cells in the PLF of septic mice induced by LPS or CLP and effect of ERβ deficiency on the pyroptosis of primary mouse PMs. (A) Flow cytometric analysis of macrophages or

neutrophils in PLF from WT and KO septic mice subjected to intraperitoneal injection of LPS. n=6-7. (B) Flow cytometric analysis of TRMs in PLF from septic mice subjected to CLP. n=6. (C) Flow cytometric analysis of M1 ($CD86^{+}CD206^{-}$ cells) and M2 ($CD86^{-}CD206^{+}$ cells) macrophages in PLF from septic mice subjected to CLP. n=6. (D-G) Primary mouse PMs were treated with LPS (2 μg/mL) for 3 h, followed by ATP (5 mM) treatment for 1 h, n=3. (D) LDH release. (E) The ASC oligomerization in PMs was detected by immunofluorescence assay. (F) The interaction between NLRP3 and NEK7 in PMs was measured by using Co-IP. (G) The protein expression of NF-κB p65, p-NF-κB p65, MyD88 and TLR4 of PMs was analyzed by western blot. Unpaired student *t*-test was performed in A-G. Data are expressed as mean ± SEM. $^{\#}P < 0.05$ and $^{\#\#}P < 0.01$ *vs*. WT group. Scale bar, 5 μm.

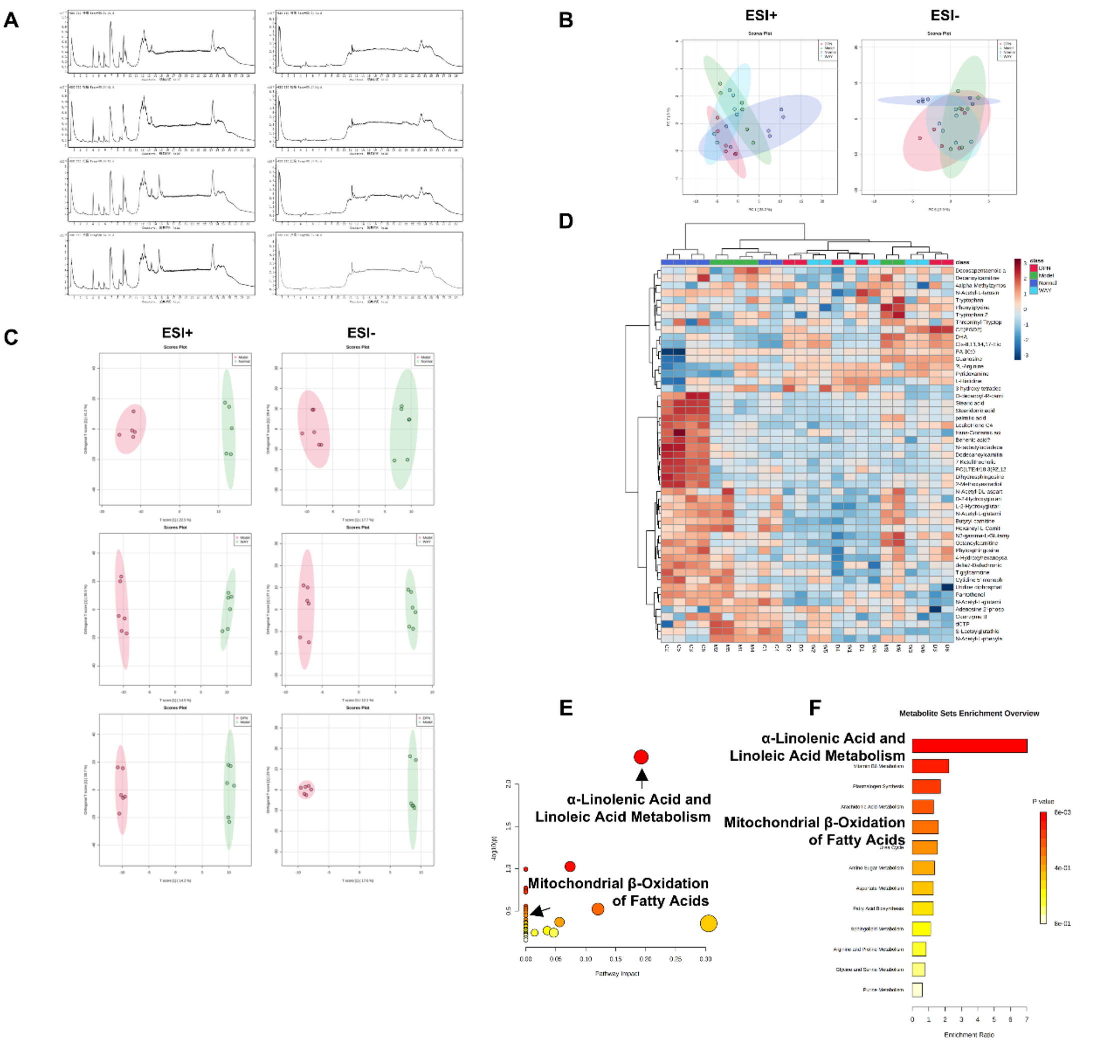

Fig. S5 Effect of ERβ activation on macrophage metabolism under pyroptosis conditions. Primary mouse PMs were stimulated with LPS (2 μg/mL), treated with or without ERβ specific agonists WAY200070 (WAY, 3 μM) or diarylpropionitrile (DPN, 3 μM) for 3 h, and then treated with ATP (5 mM). n=6. (A) The total positive and negative ion chromatograms of PMs. (B) The PCA plots of cell samples obtained from the normal group, model group, WAY (3 μM) and DPN (3 μM) groups. (C) S-plots generated from the OPLS-DA model between the normal group and model group in the ESI positive ion mode and negative ion mode. (D) Heatmap based on the relative abundance of metabolites. (E and F) Pathway analysis between Normal group and

model group.

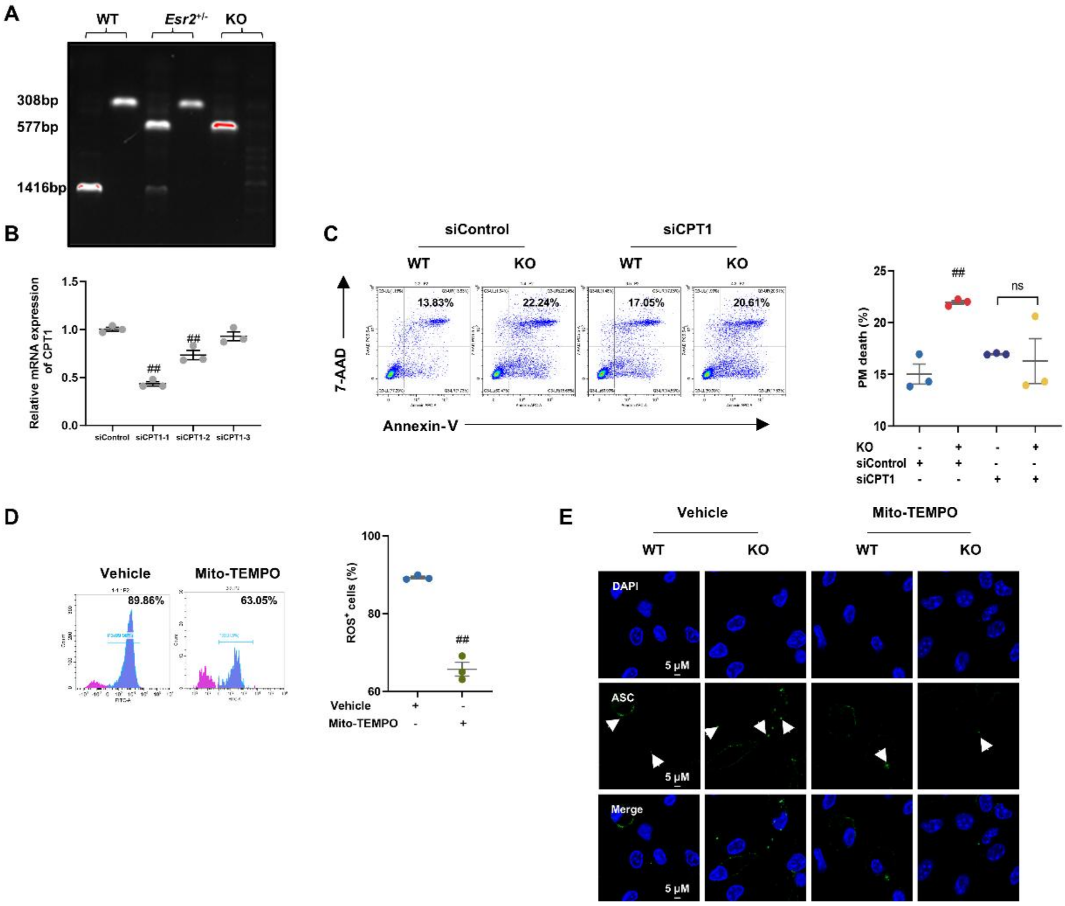

Fig. S6 Verification of gene knockout efficiency in mice and siRNA knockdown efficiency in PMs. (A) The identification of wild-type mice (WT), *Esr2*$^{+/-}$ mice, and *Esr2*$^{-/-}$ mice (KO) were analyzed by DNA gel electrophoresis. (B and C) The primary mouse PMs were transfected with siControl or siCPT1, n=3. (B) The mRNA expression was detected by using qPCR. (C) Cell death analysis of PMs. (D and E) The primary mouse PMs, pre-treated with mitochondrial-specific ROS inhibitor Mito-TEMPO (50 nM, HY-112879, MCE) or vehicle for 24 h, were treated with LPS (2 μg/mL) for 3 h, followed by ATP (5 mM) treatment for 1 h, n=3. The proportion of ROS-positive cells was quantified by flow cytometry. (E) The display diagram of ASC oligomerization in

PMs. Unpaired student *t*-test was performed in B and D. Two-way ANOVA was employed in C. Data are expressed as the means ± S.E.M. n=3 $^{\#\#}P < 0.01$ *vs.* siControl group or vehicle group.

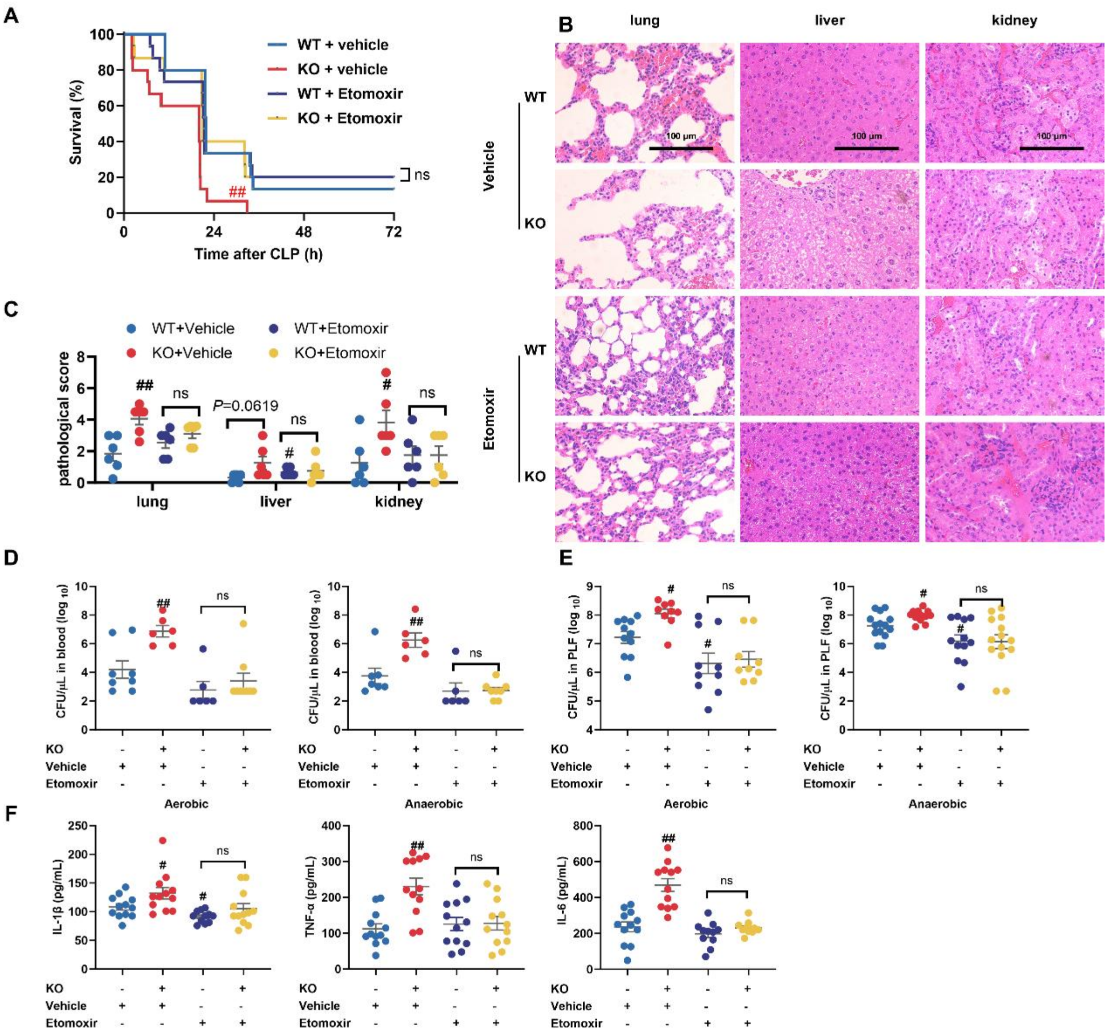

Fig. S7 Effect of etomoxir on the exacerbation of sepsis outcomes induced by CLP in the context of ERβ deficiency. Mice receiving etomoxir treatment were administered an intraperitoneal injection of 100 μL of etomoxir (3 mg/mL, Cayman Chemical) dissolved in sterile PBS containing 10% DMSO 1 h prior to the establishment of the CLP model. (A) The survival curve of septic mice, n = 15. (B and

C) H&E staining and histopathological scores of the lung, liver and kidney from septic mice. n=6. (D and E) Quantification of bacterial colonies in the blood and PLF from septic mice. n = 6-14. (F) Quantification of cytokines in the sera from septic mice. n = 11-12. Long rank (Mantel Cox) test was adopted to compare the significance in A. Two-way ANOVA was employed in C-F. Data are presented as mean ± SEM. $^{\#}P < 0.05$ and $^{\#\#}P < 0.01$ *vs*. WT + control group. ns, no significance. Scale bar, 100 μm.

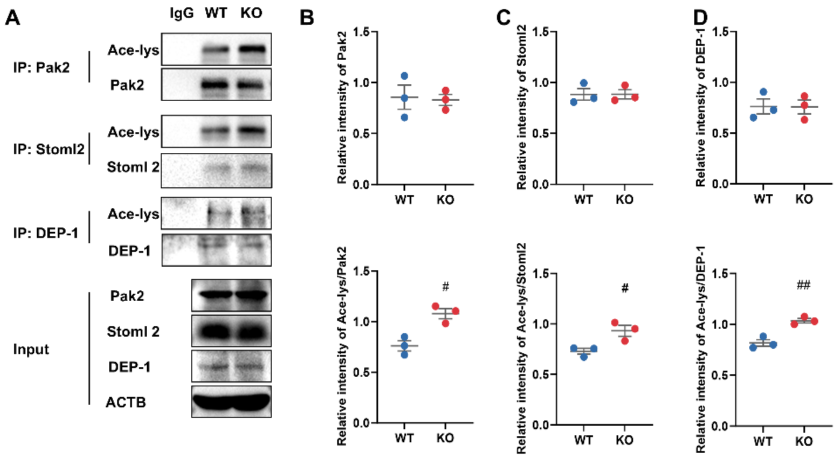

Fig. S8 Effect of ERβ deficiency on the acetylation of lysine of pak2, stoml2 and DEP-1. (A-D) The primary mouse PMs were treated with LPS (2 μg/mL) for 3 h, followed by ATP (5 mM) treatment for 1 h, n=3. The acetylation of lysine of Pak2, Stoml2 and DEP-1 in PMs was measured by using CO-IP. Unpaired student *t*-test was performed in B-D. Data are expressed as the means ± S.E.M. n = 3. $^{\#}P < 0.05$ and $^{\#\#}P < 0.01$ *vs*. WT group.

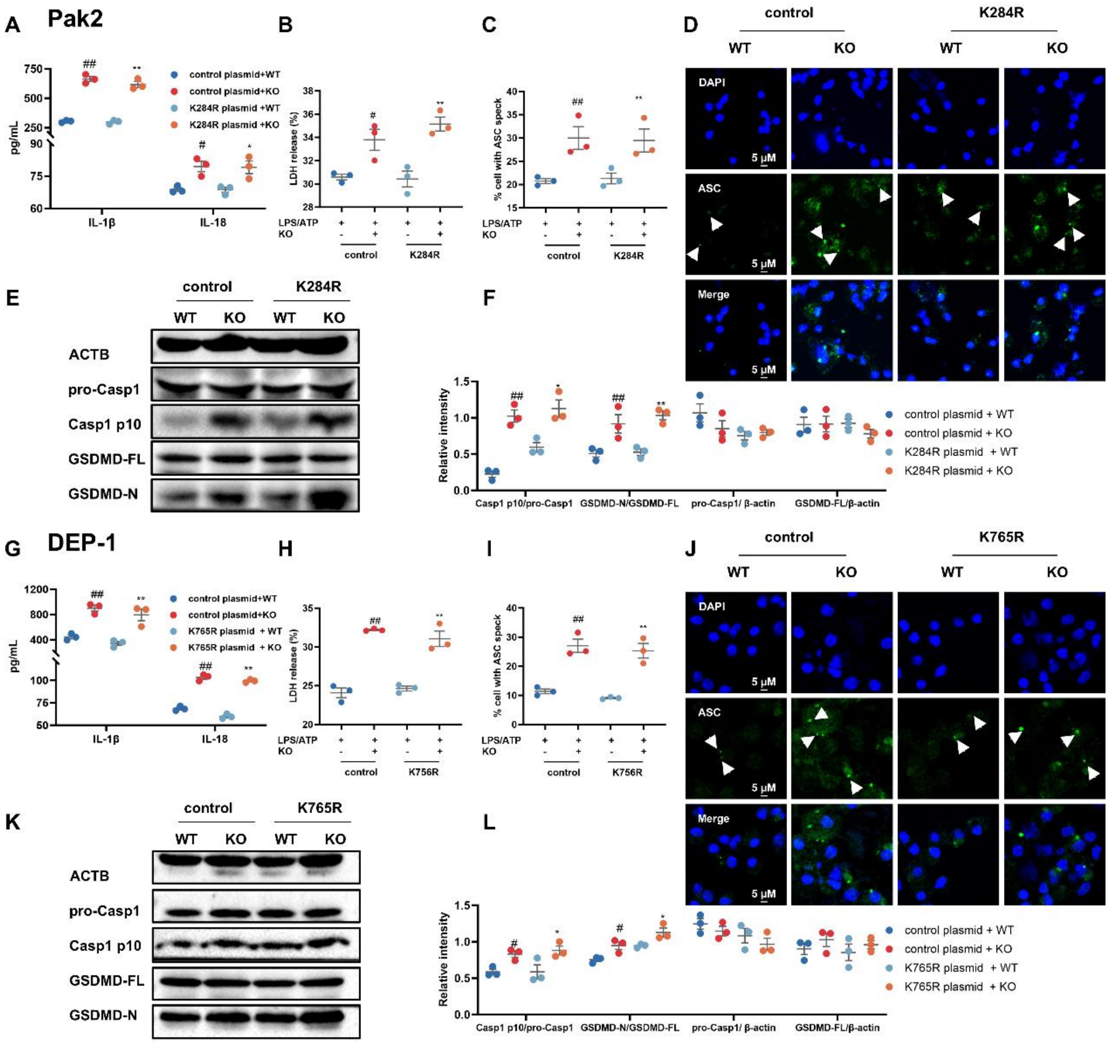

Fig. S9 Effect of mutations in the acetylated lysine site of Pak2 or DEP-1 on ERβ deficiency-drove macrophage pyroptosis. (A-F) The primary mouse PMs, transfected with either control plasmid or K284R plasmid, were treated with LPS (2 μg/mL) for 3 h, followed by ATP (5 mM) treatment for 1 h, n=3. (A) IL-1β and IL-18 levels, (B) LDH release were measured in the supernatants of PMs. (C and D) The ASC oligomerization in PMs was measured by using immunofluorescence assay. (E and F) The protein expression of Casp1 p10, Pro-Casp1, GSDMD-FL and GSDMD-N of PMs was analyzed by western blot. (G-L) The primary mouse PMs, transfected with either control plasmid or K765R plasmid, were treated with LPS (2 μg/mL) for 3 h, followed

by ATP (5 mM) treatment for 1 h, n=3. (G) IL-1β and IL-18 levels, (H) LDH release were measured in the supernatants of PMs. (I and J) The ASC oligomerization in PMs was measured by using immunofluorescence assay. (K and L) The protein expression of Casp1 p10, Pro-Casp1, GSDMD-FL and GSDMD-N of PMs was analyzed by western blot. Two-way ANOVA was employed in A-C, F, G-I and L. Data are presented as mean ± S.E.M. n = 3. $^{\#}P < 0.05$ and $^{\#\#}P < 0.01$ *vs.* control plasmid + WT group; $^{*}P < 0.05$ and $^{**}P < 0.01$ *vs.* K284R plasmid + WT group or K765R plasmid + WT group. Scale bars: 5 μm.

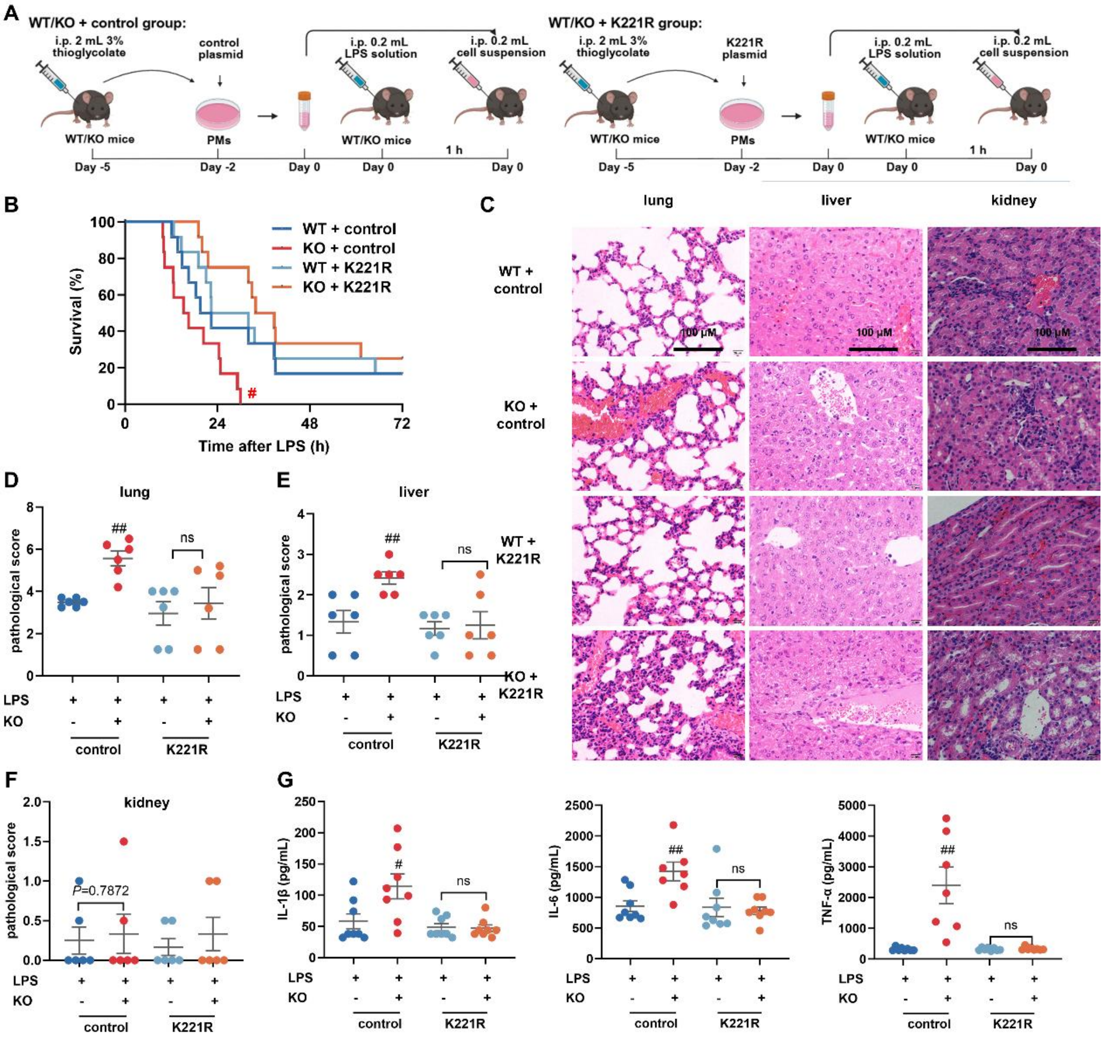

Fig. S10 Effect of mutations in the acetylated lysine site of Stoml2 on the exacerbation of sepsis outcomes induced by LPS in the context of ERβ deficiency. (A) The study protocol is shown. Adoptive transfer of PMs with the Stoml2 K221 point mutation into corresponding LPS-induced sepsis mice. (B) The survival curve of septic mice, n = 12. (C-F) H&E staining and histopathological score of the lung, liver and kidney from septic mice. n=6. (G) Quantification of cytokine levels in the sera from septic mice. n=7-8. Long rank (Mantel Cox) test was adopted to compare the significance in B. Two-way ANOVA was employed in D-G. Data are expressed as mean ± SEM. $^{\#}P < 0.05$ and $^{\#\#}P < 0.01$ *vs*. WT + control group. ns, no significance. Scale bar, 100 μm.

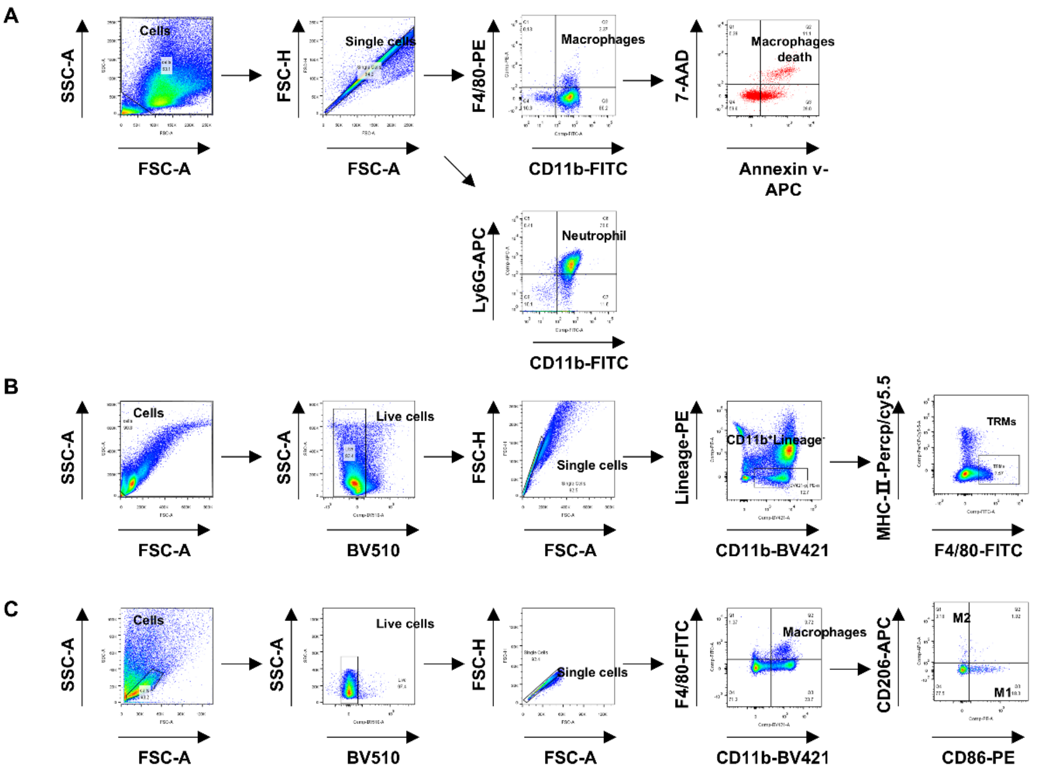

Fig. S11 Gating strategies used for flow cytometry. (A) Gating strategy to analyze PMs, peritoneal neutrophils, and the proportion of macrophage death in experimental

models. (B) Gating strategy to analyze TRMs in the PLF of septic mice. Lineage (Ly6G CD19 CD90.2)(26). (C) Gating strategy to analyze M1 and M2 in the PLF of septic mice.

Table. S1 Patients Characteristics

Demographics	Sepsis			Non sepsis
	All patients	Patients with high ERβ expression	Patients with low ERβ expression	
Age (years, mean ± SEM)	76.30 ± 13.79	89.33 ± 7.17	75.42 ± 13.16	45.36 ± 24.29
Male (n, %)	18, 69.23 %	6, 100%	12, 100%	12, 40%
**Source of infection**				
≥2 (n, %)	10, 38.46 %	2, 33.33%	3, 25.00%	
Abdominal (n, %)	2, 7.69 %	1, 16.67%	0, 0.00%	
Pulmonary (n, %)	25, 92.31 %	4, 66.67%	12, 100%	
Urinary (n, %)	7, 26.92 %	1, 16.67%	2, 16.67%	
Soft tissue (n, %)	2, 7.69 %	1, 16.67%	1, 8.33%	
Others (n, %)	1, 3.84 %	1, 16.67%	0, 0.00%	
**Comorbidities**				
≥2 (n, %)	16, 61.54 %	5, 83.33%	7, 58.33%	
None (n, %)	2, 7.69 %	0, 0.00%	1, 8.33%	
Diabetes (n, %)	7, 26.92 %	2, 33.33%	1, 8.33%	
Trauma/Surgery (n, %)	3, 11.54 %	1, 16.67%	2, 16.67%	
Cancer (n, %)	9, 34.62 %	2, 33.33%	4, 33.33%	

Heart failure (n, %)	16, 61.54 %	4, 66.67%	8, 66.67%
Kidney disease (n, %)	13, 50.00 %	5, 83.33%	4, 33.33%
**Disease scores**			
PCT (ng/ml)	6.14 ± 11.94	1.57 ± 2.24	10.09 ± 16.91
CRP (mg/L)	93.00 ± 74.03	87.40 ± 72.01	116.98 ± 81.95
SOFA	9.62 ± 4.02	7.33 ± 2.42	11.5 ± 3.48
**28-day mortality rate**			
	9, 34.62%	2, 33.33%	6, 50%
**Complete blood count (CBC) data**			
Neutrophil percentage (%, mean ± SEM)	83.00 ± 11.64	86.05 ± 4.30	86.58 ± 11.39
Lymphocyte percentage (%, mean ± SEM)	10.40 ± 10.23	6.2 ± 2.73	8.33 ±10.17
Mononuclear macrophage percentage (%, mean ± SEM)	5.67 ± 2.56	6.83 ± 1.73	4.19 ± 2.67
Eosinophil percentage (%, mean ± SEM)	0.73 ± 1.78	0.63 ± 1.06	0.75 ± 2.47

Basophil percentage (%, mean ± SEM)	0.20 ± 0.22	0.28 ± 0.24	0.15 ± 0.09

Table. S2 Human primer pairs used in real-time PCR

Gene	Sequence (5’-3’)	Length (bp)
ESR2	Forward	CCTTAATTCTCCTTCCTCCTACAACTG
	Reverse	CTGGATATTCATGGTGGCTGTCTAC
ENPP2	Forward	GTGGACCAATCTTCGACTATGACTATG
	Reverse	GGTGATGATGCTGTAGTAGTGAGTTG
CAV1	Forward	GCATTTACTTCGCCATTCTCTCTTTC
	Reverse	GTAGATGGAATAGACACGGCTGATG
ESR1	Forward	CTCCTCATCCTCTCCCACATCAG
	Reverse	AGCATCTCCAGCAGCAGGTC
BRCAa1	Forward	ACCGTTGCTACCGAGTGTCTG
	Reverse	TGATGTTCCTGAGATGCCTTTGC
TFF1	Forward	GCCGAGGCCCAGACAGAG
	Reverse	GTCGAAACAGCAGCCCTTATTTG
GPER1r	Forward	CTCTCGGAGCACCAGCAGTAC
	Reverse	TCACCACCAGGATCAGGATGTTG
ACTB	Forward	CATGTACGTTGCTATCCAGGC
	Reverse	CTCCTTAATGTCACGCACGAT

Table. S3 Mouse primer pairs used in real-time PCR

Gene	Sequence (5’-3’)	Length (bp)
Cpt1	Forward	CTACATCACCCCAACCCATATT
	Reverse	GATCCCAGAAGACGAATAGGTT
Actb	Forward	AGCAAGCAGGAGTACGATGAG
	Reverse	GGTGTAAAACGCAGCTCAGTAA

Supplemental Table 4

REAGENT or RESOURCE	SOURCE	IDENTIFIER
**Antibodies**		
Recombinant anti-pro caspase-1 (pro-Casp1) + p10 (Casp1 p10) + p12 antibody	Abcam	Cat# ab179515
APC anti-mouse Ly6G antibody	Biolegend	Cat# 127613
Alexa Fluor® 488 anti-mouse/human CD11b antibody	Biolegend	Cat# 101217
Bv421 anti-mouse/human CD11b antibody	Biolegend	Cat# 101235
PE anti-mouse F4/80 antibody	Biolegend	Cat# 123110
FITC anti-mouse F4/80 antibody	Biolegend	Cat# 123107
PerCP/Cyanine5.5 anti-mouse I-A/I-E antibody	Biolegend	Cat# 107626
PE anti-mouse Ly6G antibody	Biolegend	Cat# 127608
PE anti-mouse CD19 antibody	Biolegend	Cat# 115507
PE anti-mouse CD90.2 antibody	Biolegend	Cat# 105307
PE-conjugated anti-mouse CD86	Biolegend	Cat# 105007

APC-conjugated anti-mouse CD206 antibody	Biolegend	Cat# 141707
Acetyl-histone H3 monoclonal antibody	Bioworld	Cat# MB65867
Gasdermin D (GSDMD-FL) rabbit mAb antibody	CST	Cat# 39754
Cleaved gasdermin D (GSDMD-N) rabbit mAb antibody	CST	Cat# 36425
β-actin rabbit mAb antibody	CST	Cat# 4970
NLRP3 rabbit mAb antibody	CST	Cat# D4D8T
NF-κB p65 mouse antibody	Proteintech	Cat# 66535-1-Ig
p-NF-κB p65 mouse antibody	Proteintech	Cat# 82335-1-RR
TLR4 mouse antibody	Proteintech	Cat# 66350-1-Ig
ASC mouse antibody	Santa	Cat# sc-22514-R
NIMA Related Kinase 7 (NEK7) mouse antibody	Santa	Cat# sc-393539
CPT1 mouse antibody	Santa	Cat# sc-514555
PAK2 mouse antibody	Santa	Cat# sc-373740
Stoml2 mouse antibody	Santa	Cat# sc-376869
DEP-1 mouse antibody	Santa	Cat# sc-390404
MyD88 mouse antibody	Wanleibio	Cat# WL02494
**Biological Samples**		

Septic patient samples	This study	See Table. S1
**Chemicals**		
NP-40 buffer	Beyotime	Cat# P0013F
Phenylmethanesulfonyl fluoride	Beyotime	Cat# ST505
Lipo6000	Beyotime	Cat# C0526
Blood agar plate medium	Bikeman	Cat# 110701019
VX765	MCE	Cat# HY-13205
Necrostatin-1	MCE	Cat# HY-15760
Z-DEVD-FMK	MCE	Cat# HY-12466
Ferrostatin-1	MCE	Cat# HY-100579
ATP	MCE	Cat# HY-B2176
Protein A/G magnetic beads	MCE	Cat# HY-K0202
Mito-TEMPO	MCE	Cat# HY-112879
2-DG	MCE	Cat# HY-13966
Mouse GM-CSF recombinant protein	PeproTech	Cat# 315-03
Isoflurane	RWD	Cat# R510-22-10
LPS	Sigma	Cat# L2880
Thioglycolate	Sigma	Cat# 106691
Diamidino-2-phenylindole (DAPI)	Sigma	Cat# 10236276001
**Critical Commercial Assays**		
Seahorse XF palmitate oxidation stress test kit	Agilent	Cat# 103693-100

Seahorse XF Glycolytic Rate Assay kit	Agilent	Cat# 103344-100
BCA protein colorimetric assay kit	Beyotime	Cat# P0010S
LDH cytotoxicity assay kit	Beyotime	Cat# C0016
MMP assay kit	Beyotime	Cat# C2006
Reactive oxygen species assay kit	Beyotime	Cat# S0033S
Acetyl-CoA ELISA kit	mlbio	Cat# YJ258897
Mouse IL-18 ELISA kit	MultiSciences Biotech	Cat# EK218
Annexin V-APC/7-AAD apoptosis kit	MultiSciences Biotech	Cat# AP105
Pyruvate assay kit	njjcbio	Cat# A081-1-1
PDH activity assay kit	Solarbio	Cat# BC0385
Mouse IL-1β uncoated ELISA kit	ThermoFisher	Cat# 88-7013A-88
Mouse IL-6 uncoated ELISA kit	ThermoFisher	Cat# 88-7064-88
Mouse TNF alpha uncoated ELISA kit	ThermoFisher	Cat# 88-7324-76
Mouse IL-10 uncoated ELISA kit	ThermoFisher	Cat# 88-7105-88
**Experimental Models:**		
Esr2 (the gene encoding ERβ) KO mice	Shanghai Model Organisms Center, Inc.	N/A
**Oligonucleotides**		

Primers for qPCR	This study	See Table. S2 and Table. S3
si-CPT1-1 CCAAAUUGCAGUGGUAUUUTT	This study	N/A
si-CPT1-2 CCUGCAUUCCUUCCCAUUUTT	This study	N/A
si-CPT1-3 CCACCUCUUCUGCCUCUAUTT	This study	N/A
**Recombinant DNA**		
Pak2 plasmid	This study	N/A
Pak2 K284R plasmid	This study	N/A
Stoml2 plasmid	This study	N/A
Stoml2 K221R plasmid	This study	N/A
DEP-1 plasmid	This study	N/A
DEP-1 K765R plasmid	This study	N/A
**Deposited data**		
Non-target metabolomics data	This study	OMIX014575
Quantitative acetylome analysis data	This study	PXD073393
**Software**		
ImageJ software	NIH	https://imagej.nih.gov/ij/

Graphpad Prism 8 software	GraphPad	https://www.graphpad.com/scientific-software/prism/
FlowJo V10	BD	https://www.flowjo.com/
ZEN lite 2.6 software	Zeiss	https://www.zeiss.com/
