# Supplementary material for: Estrogen receptor β deficiency increases susceptibility to sepsis through metabolic reprogramming–induced macrophage pyroptosis
Source: J Clin Invest. 2026 Mar 17;136(10):e196636. doi: 10.1172/JCI196636 (PMC13178654; doi:10.1172/JCI196636)
Supplement: Unedited blot and gel images [file jci-136-196636-s280.pdf]

Fig. 3H

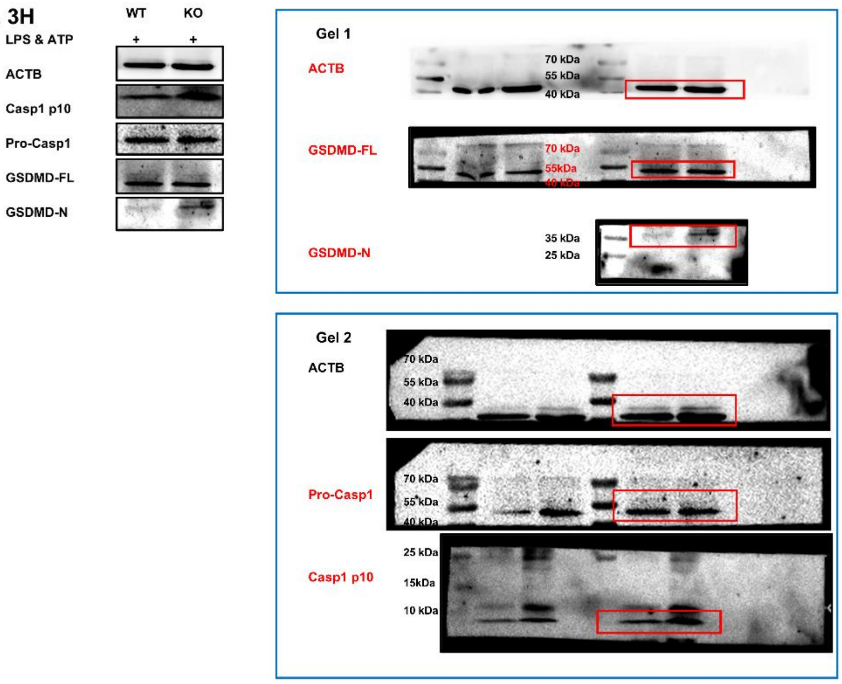

Fig. 4C

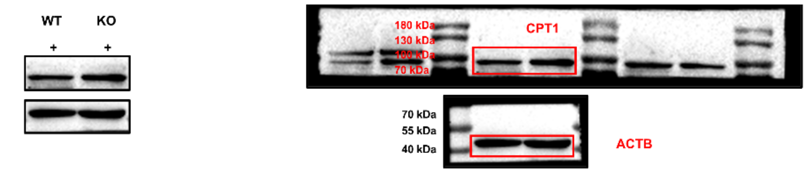

Fig. 4L

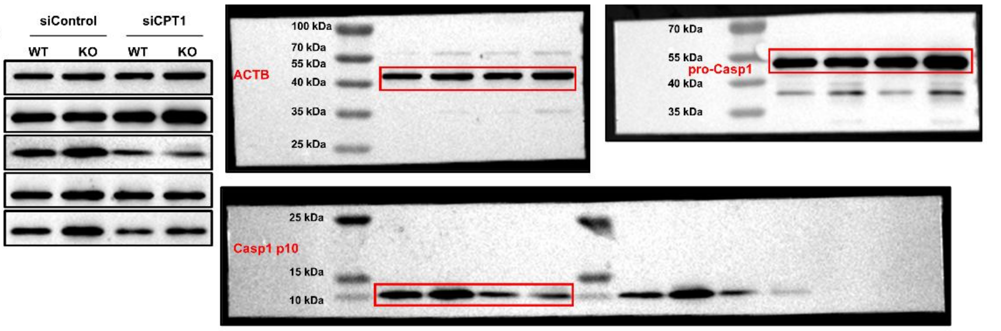

The same gel with different exposure times

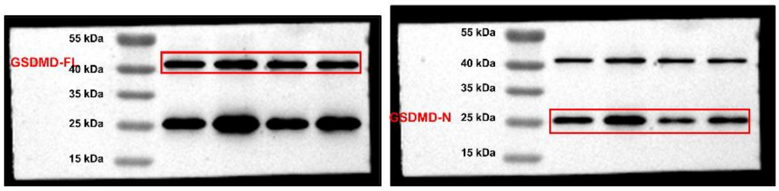

Fig. 5H

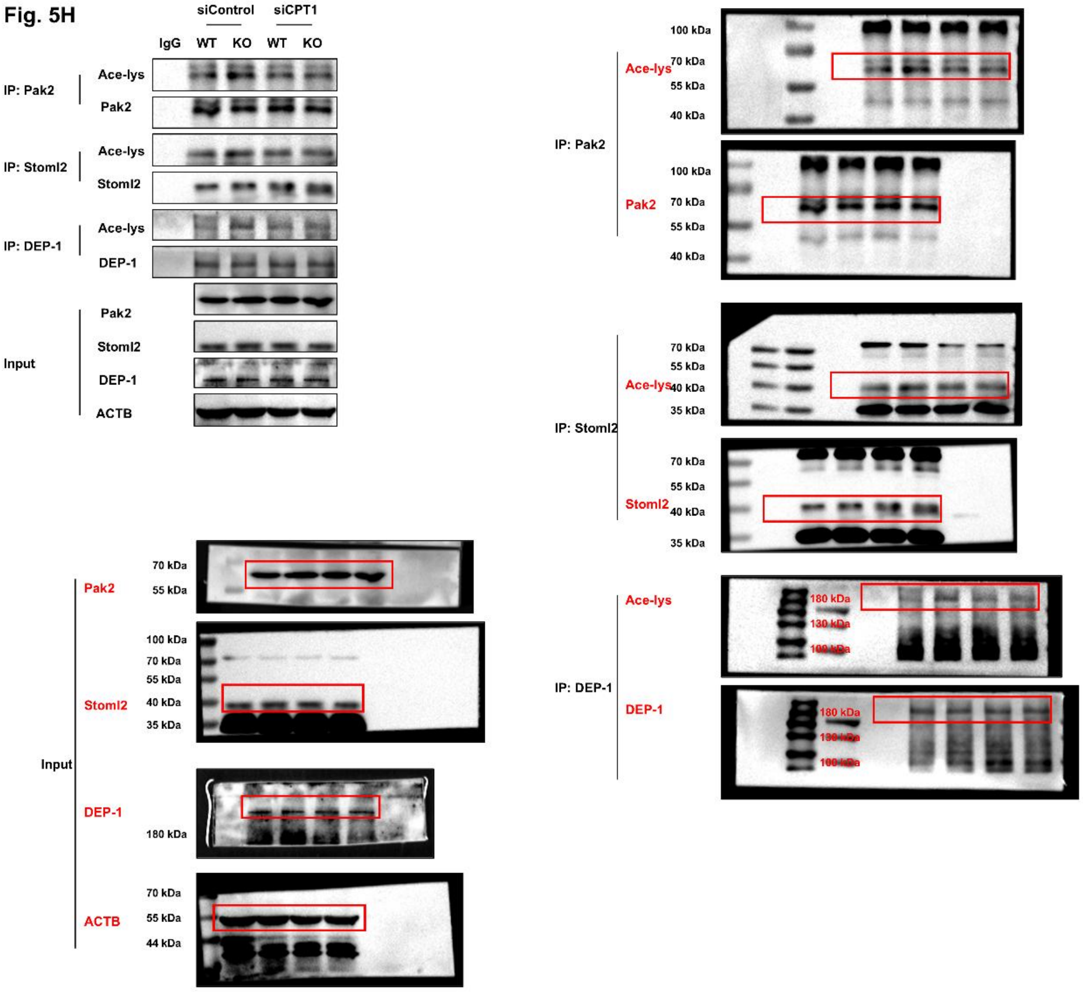
siControl
siCPT1
IgG
WT
KO
WT
KO
IP: Pak2
Ace-lys
Pak2
IP: Stoml2
Ace-lys
Stoml2
IP: DEP-1
Ace-lys
DEP-1
Input
Pak2
Stoml2
DEP-1
ACTB
100 kDa
70 kDa
55 kDa
40 kDa
35 kDa
180 kDa
130 kDa
100 kDa
44 kDa

Fig. 6E

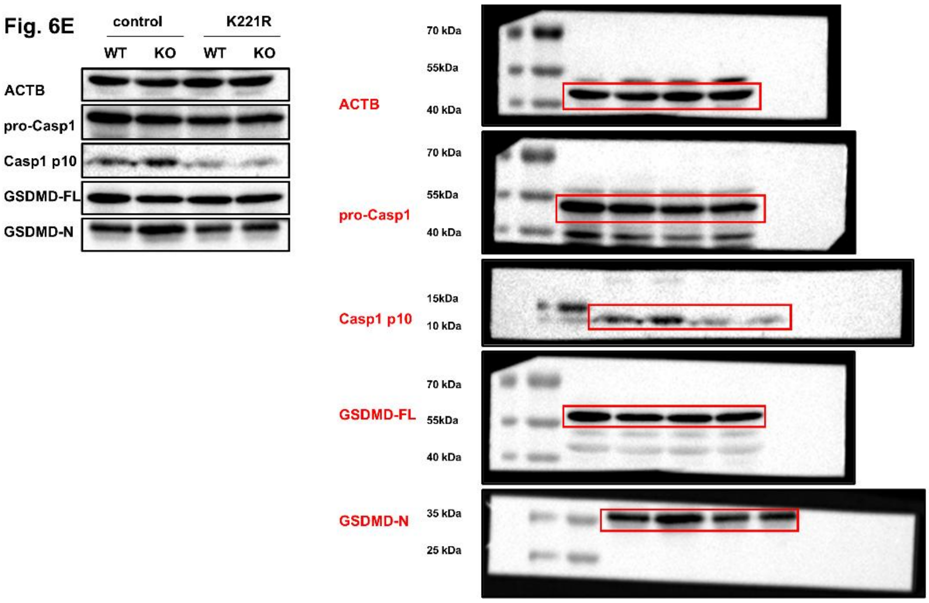
control
K221R
WT
KO
WT
KO
ACTB
pro-Casp1
Casp1 p10
GSDMD-FL
GSDMD-N
70 kDa
55kDa
40 kDa
15kDa
10 kDa
35 kDa
25 kDa

Fig.S4F

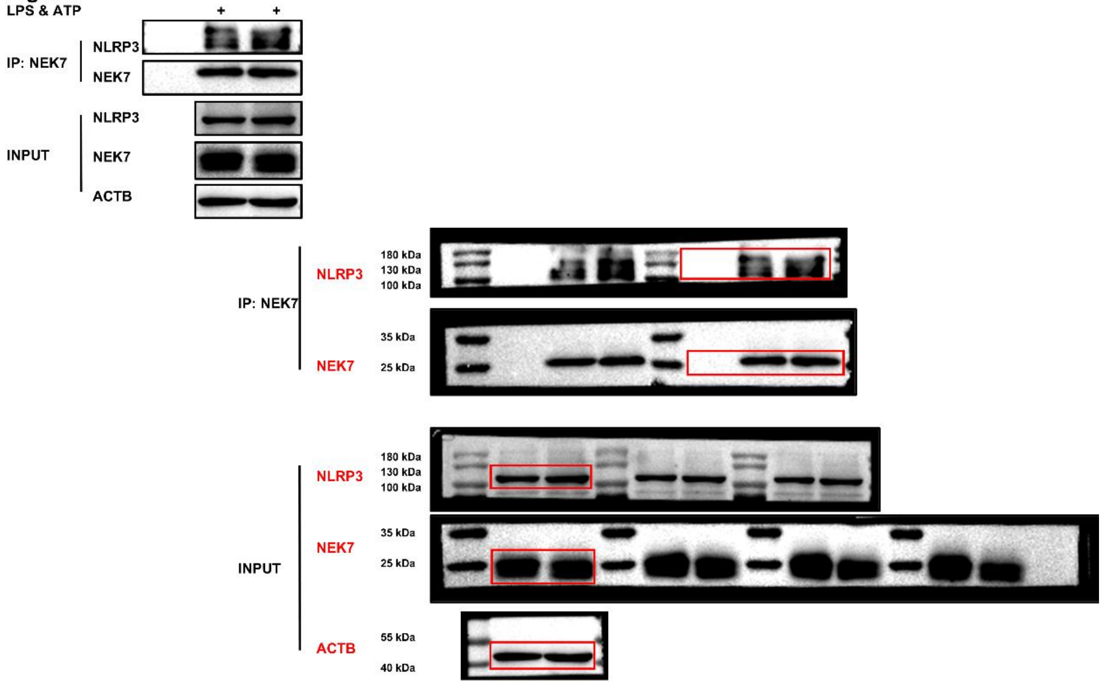

Fig.S4G

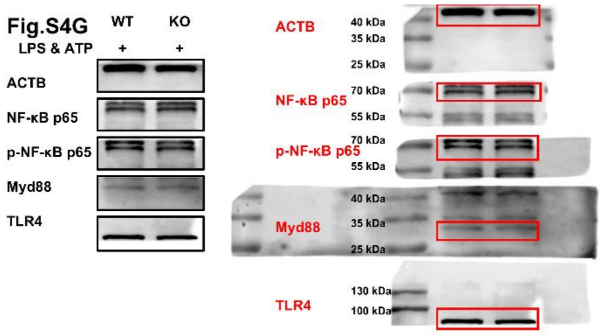

Fig.S6A

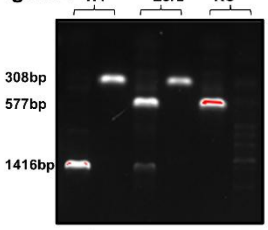

WT
Esr2+/-
KO
308bp
577bp
1416bp

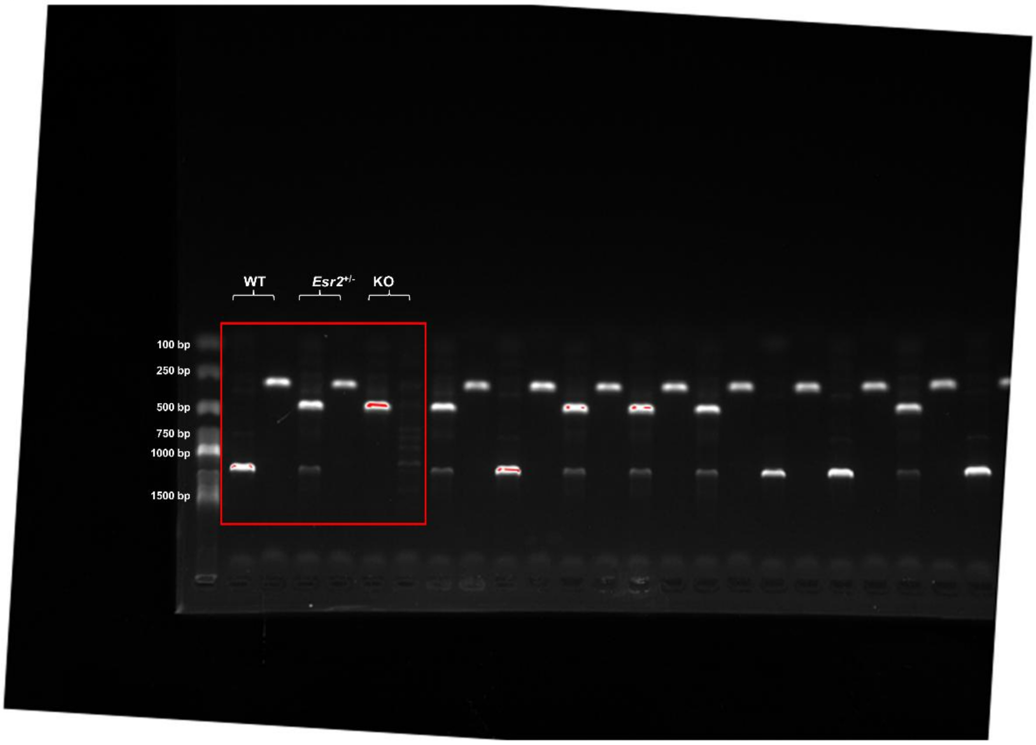

WT
Esr2+/-
KO
100 bp
250 bp
500 bp
750 bp
1000 bp
1500 bp

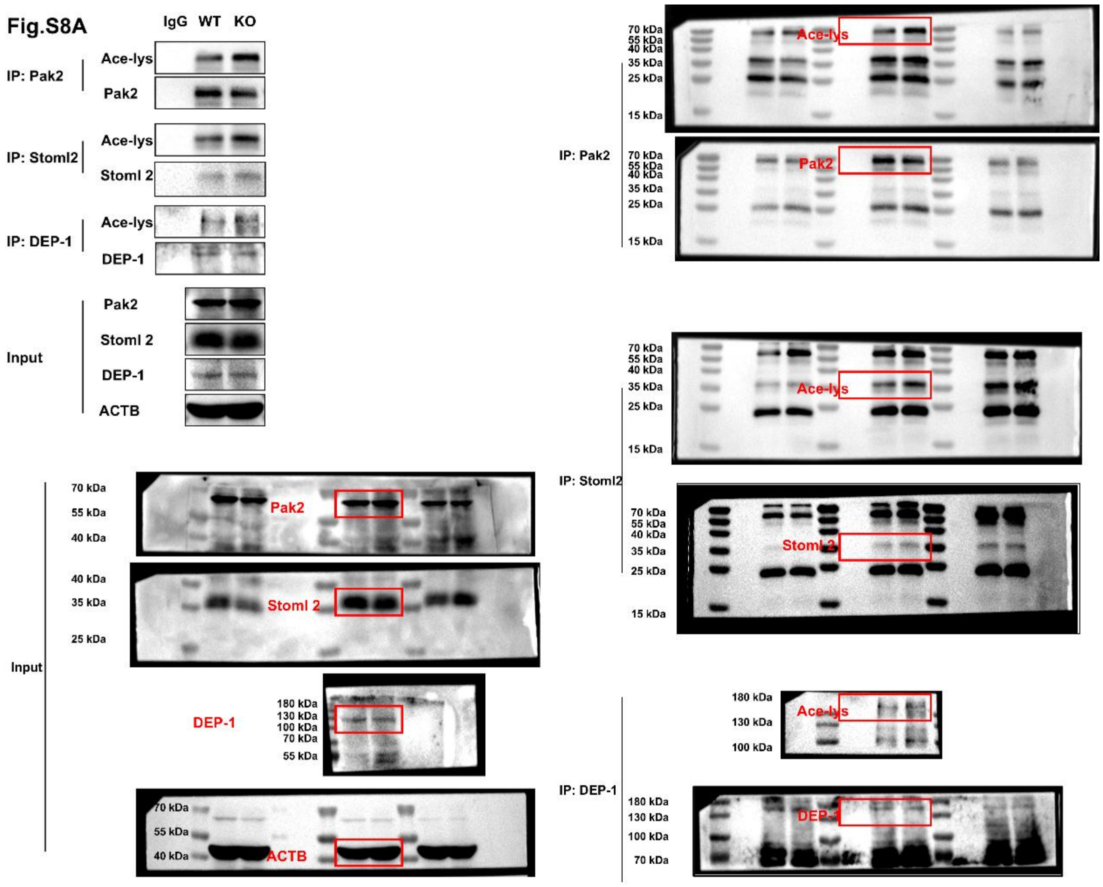

Fig.S8A
IgG WT KO
IP: Pak2
Ace-lys
Pak2
IP: Stoml2
Ace-lys
Stoml 2
IP: DEP-1
Ace-lys
DEP-1
Input
Pak2
Stoml 2
DEP-1
ACTB
70 kDa
55 kDa
40 kDa
35 kDa
25 kDa
15 kDa
180 kDa
130 kDa
100 kDa

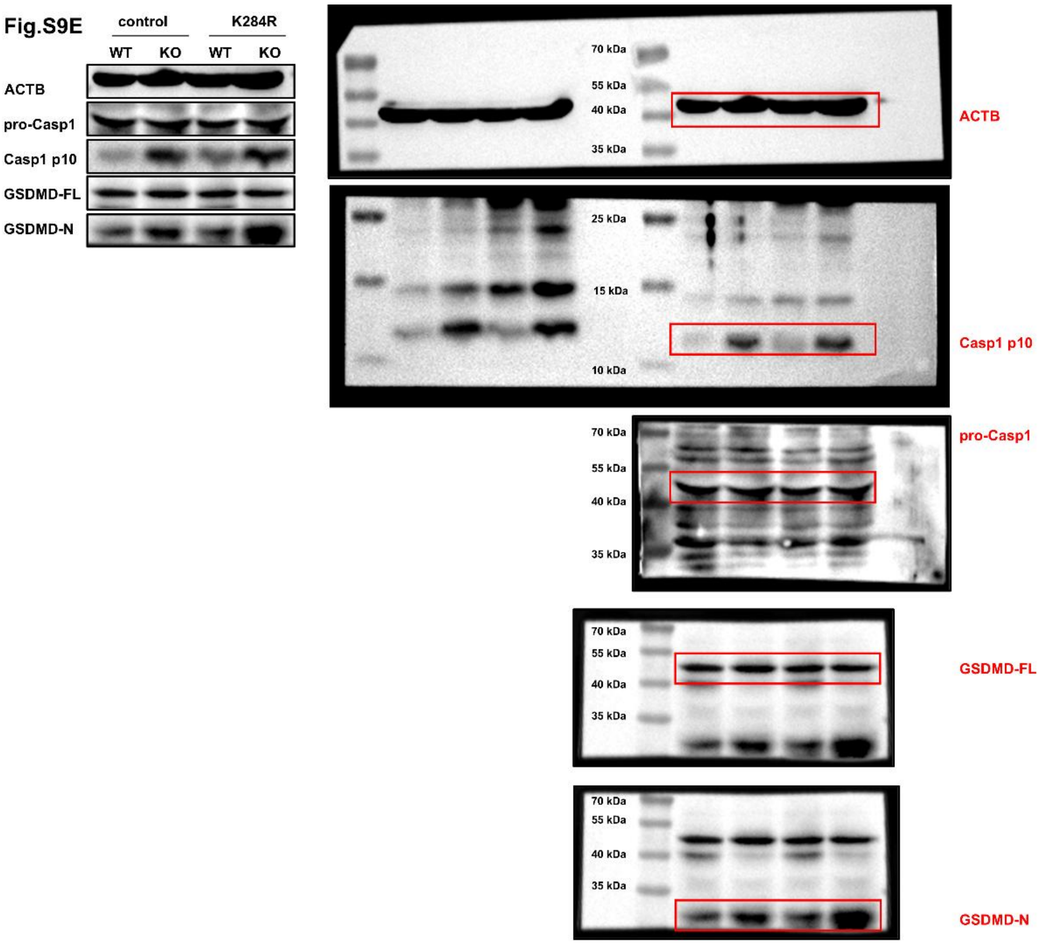
Fig.S9E
control
K284R
WT
KO
WT
KO
ACTB
pro-Casp1
Casp1 p10
GSDMD-FL
GSDMD-N
70 kDa
55 kDa
40 kDa
35 kDa
25 kDa
15 kDa
10 kDa
ACTB
Casp1 p10
pro-Casp1
GSDMD-FL
GSDMD-N

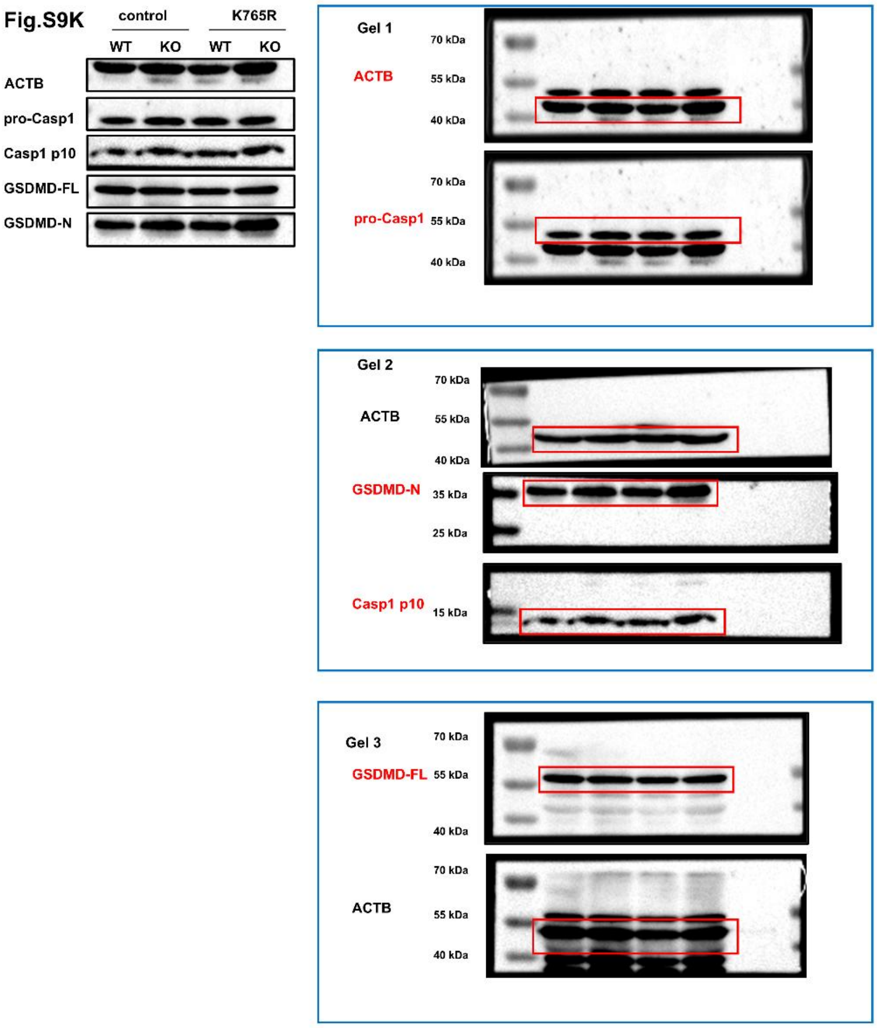

Fig.S9K
control
K765R
WT
KO
WT
KO
ACTB
pro-Casp1
Casp1 p10
GSDMD-FL
GSDMD-N
Gel 1
70 kDa
ACTB
55 kDa
40 kDa
70 kDa
pro-Casp1
55 kDa
40 kDa
Gel 2
70 kDa
ACTB
55 kDa
40 kDa
GSDMD-N
35 kDa
25 kDa
Casp1 p10
15 kDa
Gel 3
70 kDa
GSDMD-FL
55 kDa
40 kDa
70 kDa
ACTB
55 kDa
40 kDa

Fig.R4B

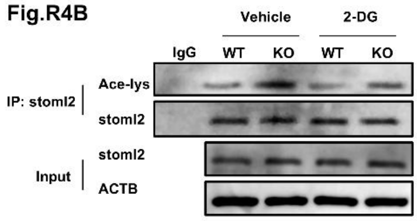
Vehicle
2-DG
IgG
WT
KO
WT
KO
Ace-lys
IP: stoml2
stoml2
stoml2
Input
ACTB

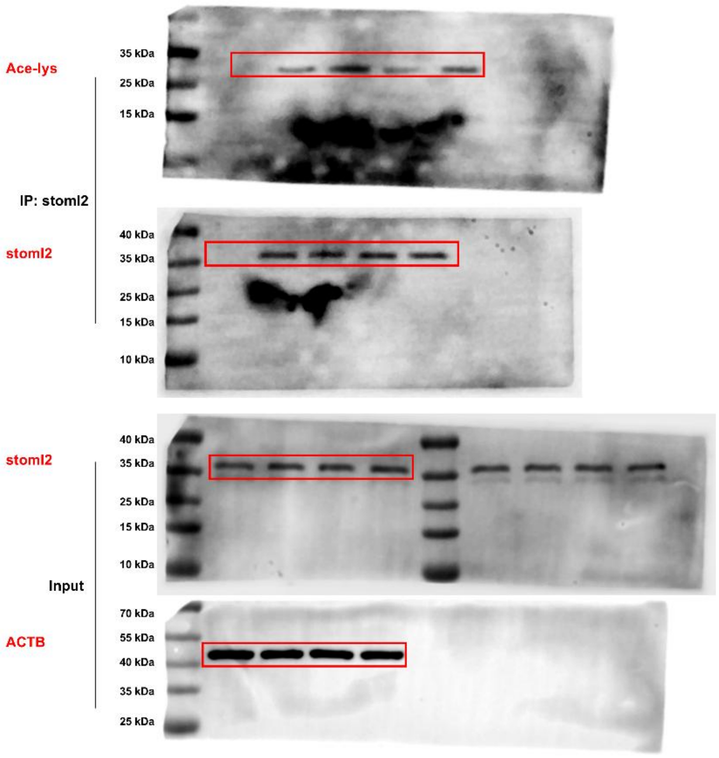
Ace-lys
35 kDa
25 kDa
15 kDa
IP: stoml2
40 kDa
stoml2
35 kDa
25 kDa
15 kDa
10 kDa
40 kDa
stoml2
35 kDa
25 kDa
15 kDa
10 kDa
Input
70 kDa
55 kDa
ACTB
40 kDa
35 kDa
25 kDa
